# Zebrafish mutants and TEAD reporters reveal essential functions for Yap and Taz in posterior cardinal vein development

**DOI:** 10.1038/s41598-018-27657-x

**Published:** 2018-07-05

**Authors:** Matteo Astone, Jason Kuan Han Lai, Sirio Dupont, Didier Y. R. Stainier, Francesco Argenton, Andrea Vettori

**Affiliations:** 1 University of Padova, Department of Biology, Padova, Italy; 2 Max Planck Institute for Heart and Lung Research, Bad Nauheim, Germany; 3 University of Padova, Department of Molecular Medicine, Padova, Italy

## Abstract

As effectors of the Hippo signaling cascade, YAP1 and TAZ are transcriptional regulators playing important roles in development, tissue homeostasis and cancer. A number of different cues, including mechanotransduction of extracellular stimuli, adhesion molecules, oncogenic signaling and metabolism modulate YAP1/TAZ nucleo-cytoplasmic shuttling. In the nucleus, YAP1/TAZ tether with the DNA binding proteins TEADs, to activate the expression of target genes that regulate proliferation, migration, cell plasticity, and cell fate. Based on responsive elements present in the human and zebrafish promoters of the YAP1/TAZ target gene *CTGF*, we established zebrafish fluorescent transgenic reporter lines of Yap1/Taz activity. These reporter lines provide an *in vivo* view of Yap1/Taz activity during development and adulthood at the whole organism level. Transgene expression was detected in many larval tissues including the otic vesicles, heart, pharyngeal arches, muscles and brain and is prominent in endothelial cells. Analysis of vascular development in *yap1/taz* zebrafish mutants revealed specific defects in posterior cardinal vein (PCV) formation, with altered expression of arterial/venous markers. The overactivation of Yap1/Taz in endothelial cells was sufficient to promote an aberrant vessel sprouting phenotype. Our findings confirm and extend the emerging role of Yap1/Taz in vascular development including angiogenesis.

## Introduction

Yes Associated Protein 1 (YAP1) and WW Domain Containing Transcription Regulator 1 (WWTR1 or TAZ), co-transcriptional effectors of Hippo signal transduction, are essential regulators of development, tissue homeostasis, and regeneration through their molecular, mechanical, and metabolic control of proliferation, migration, cell-fate, and other cellular processes^1,2^. The Hippo pathway is an evolutionarily conserved tumour suppressor signal transduction pathway culminating in YAP1/TAZ phosphorylation and inhibition. In mammals, the sterile 20-like kinases MST1/STK4 and MST2/STK3, when activated by upstream signals and bound to their regulatory protein SAV1/WW45, phosphorylate and activate the LATS1/2 kinases together with their regulatory subunits MOB1A/B^3–5^. In turn, the LATS1/2/MOB1A/B complex phosphorylates YAP1 and TAZ^6–8^ at five and four serine/threonine residues, respectively^9,10^. Active, not phosphorylated YAP1/TAZ translocate into the nucleus to drive transcription of a set of target genes as co-transcription factors of TEADs^1^.

Despite a significant wealth of knowledge on these factors, one question that remains largely unaddressed is indeed whether these factors are active in physiological conditions in many adult tissues^11–15^. Thus, the development of tools and techniques to follow YAP1/TAZ activity can be a significant step forward to address this issue.

While the role of YAP1/TAZ in organ growth and cancer is well established, the importance of their signaling in angiogenesis and vascular development has emerged only recently. Endothelium-specific inducible YAP1/TAZ knockout mice revealed that YAP1/TAZ activity is required for developmental sprouting angiogenesis and is controlled by VEGF^16^. Furthermore, YAP1/TAZ modulates actomyosin contractility for endothelial cell migration as well as establishment of tight junctions for endothelial barrier maturation^17^. In zebrafish it was demonstrated that Yap1 activation is induced by blood flow and required for vessel maintenance^18^. YAP1/TAZ expression is essential for coronary vasculature development in mouse^19^ while it has been shown that inhibition of Tead transcriptional activity can affect remodeling of the zebrafish caudal vein plexus (CVP) by vascular regression^20^. A role of YAP1/TAZ in atherosclerosis and vascular smooth muscle cell proliferation, migration and differentiation has also been described^21–27^.

The zebrafish vasculature and the basic vascular plan of its embryo are remarkably similar to that of other mammalian models^28,29^. Developmental vasculogenesis, angiogenesis and vascular remodelling recruit the same growth and differentiation factors as those involved in mammals and the physiopathology of the vascular system is also very conserved^29–31^. The zebrafish cardiovascular system develops considerably fast: a beating heart, a complete circulation loop and circulating erythroblasts are present by 24 hours post fertilization (hpf)^32,33^. In zebrafish, the dorsal aorta (DA) and posterior cardinal vein (PCV) form between the 10 somite stage (14 hpf) and 24 hpf in a process, known as vasculogenesis, that appears to be conserved among vertebrates^34,35^. From 14 hpf onward, endothelial progenitor cells located in the lateral plate mesoderm (LPM) migrate towards the midline of the embryo where they aggregate to form the DA. Shortly after the 15-somite stage, a second wave of angioblast migration follows and together with some cells from the first wave gives rise to the PCV^36^. Consequently, the angiogenic process begins with the formation of intersegmental vessels (ISVs) of the trunk that start sprouting from the DA at 20 hpf. As ISVs reach the dorsolateral surface of the neural tube, they branch caudally and rostrally, and connect with each other to form the dorsal longitudinal anastomotic vessel (DLAV)^27^.

In this work, we present a zebrafish fluorescent transgenic line that reports Yap1/Taz-Teads transcriptional activity *in vivo*. The reporter, active in many regions of embryos and larvae but largely silenced in adult fish, is prominently expressed in the cardiovascular system. To explore the role of Yap1/Taz activity in vascular development, we generated *yap1* and *taz* mutants by CRISPR/Cas9. Interestingly, *yap1*^−/−^*;taz*^+*/*−^ animals display strong alterations in PCV formation, suggesting the requirement of Yap1/Taz during the development of primary vessels.

## Results

### Generation of Yap1/Taz zebrafish reporter lines

**Figure 1:**
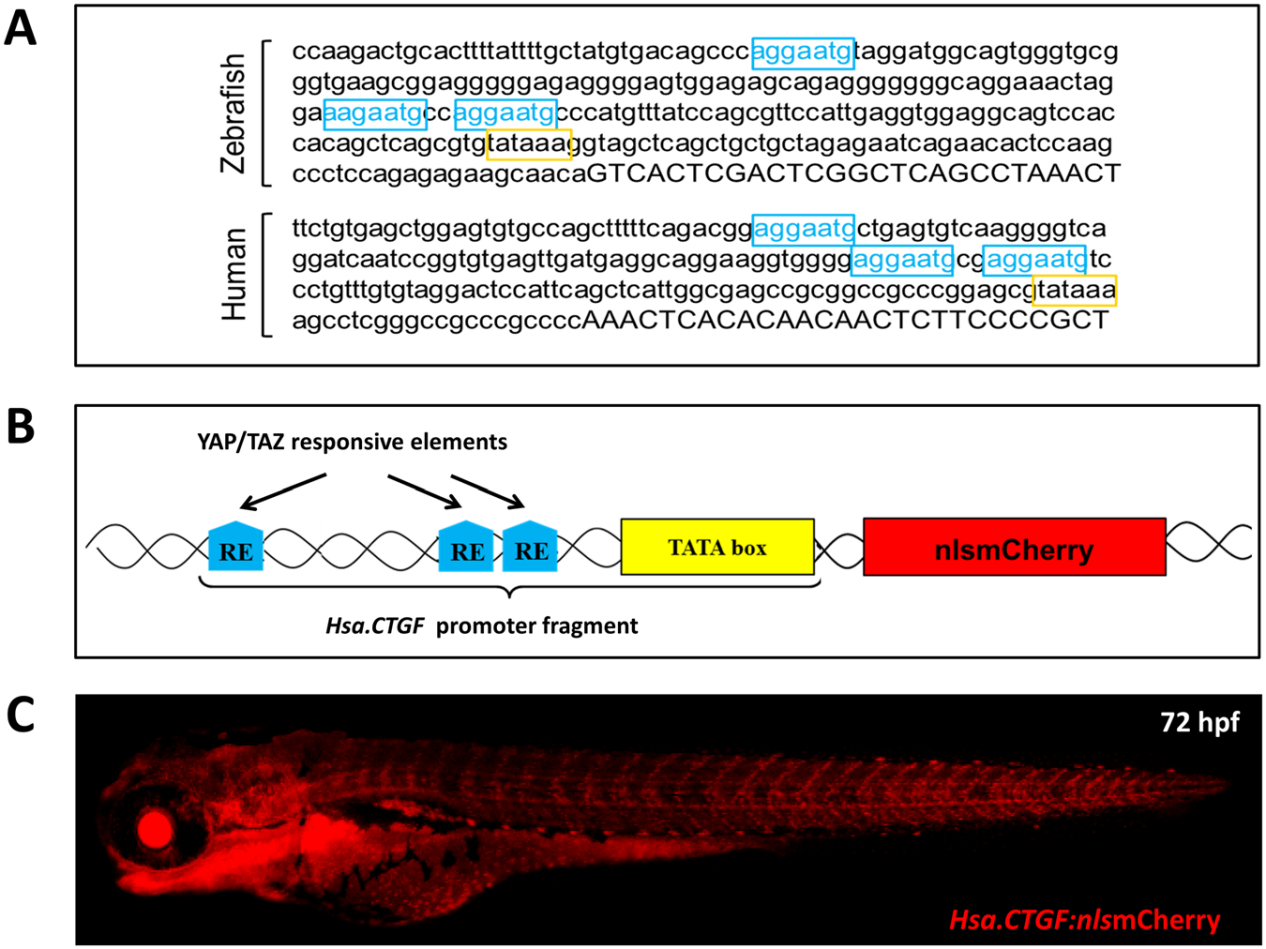
*Hsa.CTGF*-based Yap1/Taz zebrafish reporter line. (**A**) *ctgf* promoter contains three Yap1/Taz responsive elements (RE) and is remarkably conserved between zebrafish and humans. The promoter region upstream of the 5′UTR (uppercase) of the zebrafish ctgfa and human CTGF genes contain each three TEAD DNA-binding sites (YAP1/TAZ REs; in light blue) just before the TATA box (in yellow). Their sequences, orientation and distances are remarkably conserved between zebrafish and humans. (**B**) Schematic representation of the *Hsa*.*CTGF*-based Yap1/Taz reporter construct. The Yap1/Taz-responsive fragment of the construct is derived from the human *CTGF* promoter. It contains the pathway-specific REs MCAT and the TATA box, and regulates the expression of the downstream reporter gene. (**C**) Overview of a *Tg(Hsa*.*CTGF:nlsmCherry)* larva.

In order to generate a reporter to follow YAP1/TAZ activity *in vivo*, we used a YAP1/TAZ reporter construct based on the −200/+27 fragment of the human *CTGF* gene promoter (*Hsa*.*CTGF*), which contains three YAP1/TAZ/TEAD DNA-binding sites highly conserved also in the zebrafish *ctgfa* promoter^37^ (Fig. 1A). To test the responsiveness of the *Hsa*.*CTGF*-based YAP1/TAZ reporter construct, we generated a luciferase-based reporter and tested it in breast cancer cells expressing high levels of YAP1/TAZ. Transfection of the *Hsa*.*CTGF*-Lux plasmid resulted in a strong signal of the luciferase reporter gene when compared to the empty luciferase vector (not shown). Direct inhibition of YAP1/TAZ by siRNA knockdown, activation of the Hippo pathway through NF2 or treatment with latrunculin A (an F-actin inhibitor leading to YAP1/TAZ inhibition in multiple systems)^38^ were all able to inhibit the reporter expression (Fig. S1A).

The reporter construct was used to develop a *Hsa*.*CTGF*-based YAP1/TAZ zebrafish reporter. *Hsa*.*CTGF* promoter fragment was cloned into a Gateway 5′ entry clone (p5E-MCS) and used to create the *pDest(Hsa*.*CTGF:nlsmCherry)* and *pDest(Hsa*.*CTGF:eGFP))* (Fig. 1B). One-cell stage zebrafish embryos were injected with each destination vector together with Tol2 transposase mRNA. Mosaic transgenic fish displaying a strong fluorescence at 24 hpf were selected and raised to adulthood. An average of 76% (13/17) of the injected fish prescreened for mosaic fluorescence were found transmitting the transgene to their offspring. All the offspring from different founder fish exhibited a similar reporter protein expression pattern, displaying strong fluorescence in identical anatomical districts, such as the lens and otic vesicles, the heart, the pharyngeal arches and the vasculature (Figs 1C and S2). Two founders were selected to establish stable reporter lines containing a single allele in their germline: *Tg(Hsa*.*CTGF:eGFP)*^*ia48*^ and *Tg(Hsa*.*CTGF:nlsmCherry)*^*ia49*^.

### *Hsa*.*CTGF*-based zebrafish transgenic lines are *bona fide* Yap1/Taz reporter

**Figure 2:**
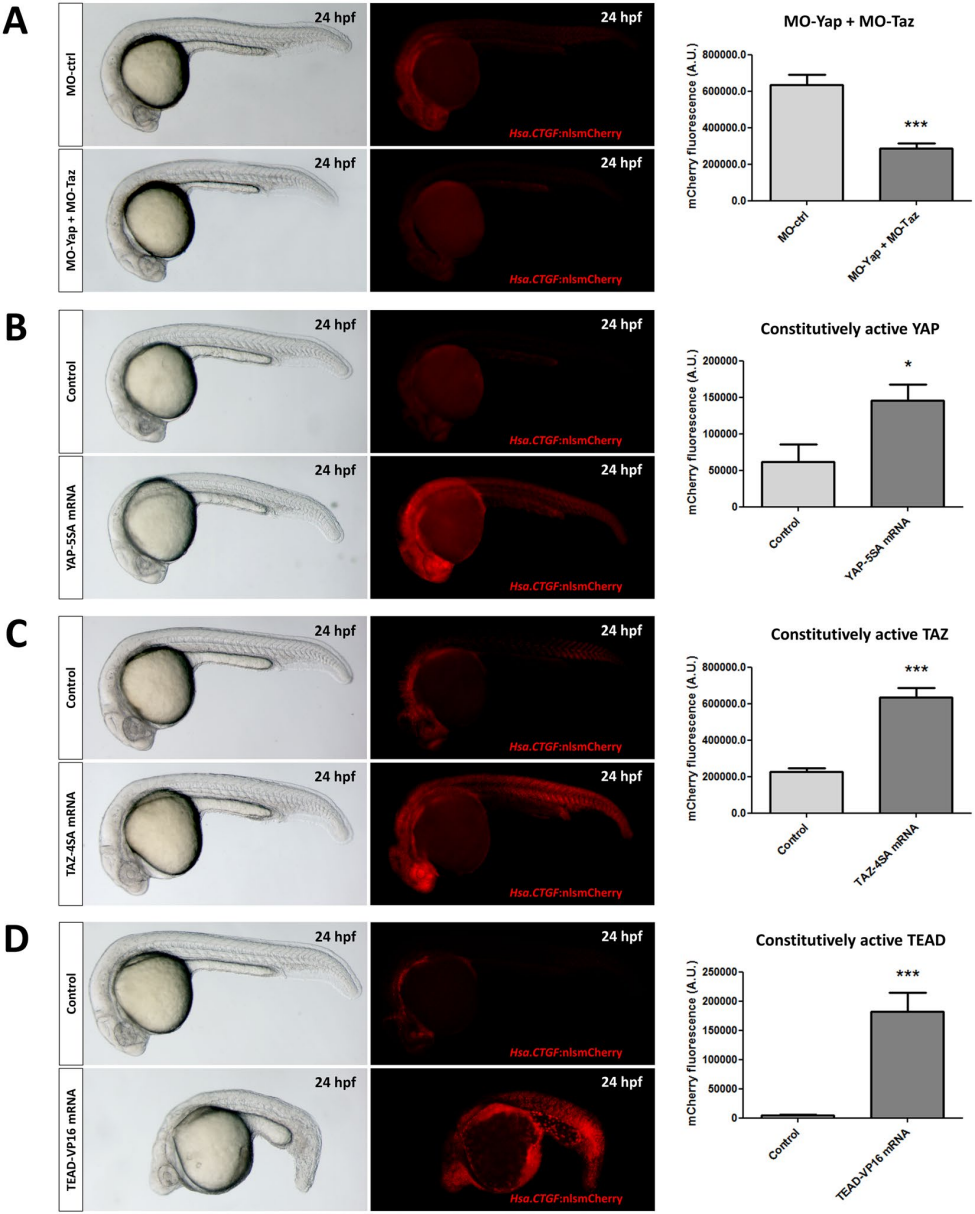
*Hsa.CTGF*-based zebrafish transgenic lines are *bona fide* Yap1/Taz reporter. (**A**) Morpholino-mediated Yap1/Taz knockdown reduces *Hsa*.*CTGF* reporter signal (in red). Two splice morpholinos, targeting respectively Yap1 and Taz pre-mRNAs, were co-injected in one-cell stage *Tg(Hsa*.*CTGF:nlsmCherry)*^*ia49*^ embryos. (**B**–**D**) Constitutive activation of Yap1/Taz-mediated transcription increases *Hsa*.*CTGF* reporter signal (in red). The mRNA coding for constitutively active versions of YAP1 (**B**), TAZ (**C**) or TEAD (**D**) were injected in one-cell stage *Tg(Hsa*.*CTGF:nlsmCherry)*^*ia49*^ embryos. The fluorescent reporter expression was documented and quantified at 24 hpf. Control morpholino-injected: n = 20; Yap/Taz morpholino-injected: n = 29; controls for YAP-5SA: n = 12; YAP-5SA mRNA-injected: n = 12; controls for TAZ-4SA: n = 57; TAZ-4SA mRNA-injected: n = 56; controls for TEAD-VP16: n = 20; TEAD-VP16 mRNA-injected: n = 20. A.U.: arbitrary units; *p < 0.05; ***p < 0.001.

To validate whether the *Hsa*.*CTGF*-based zebrafish reporter lines can be used to visualize the endogenous activity of YAP1/TAZ *in vivo*, we knocked down zygotic expression of both *yap1* and *taz* by co-injecting one-cell stage embryos with two splice morpholinos targeting respectively *yap1* and *taz* pre-mRNAs. mCherry expression of the *Tg(Hsa*.*CTGF:nlsmCherry)*^*ia49*^ reporter line was significantly reduced throughout the entire embryo compared to control morpholino-injected embryos (Fig. 2A). The knockdown of endogenous Yap1 and Taz proteins was confirmed by Western blotting using whole embryo extracts (Fig. S1B).

To test whether the reporter line was able to visualize increased YAP1/TAZ activities, we injected in one-cell stage embryos of the *Tg(Hsa*.*CTGF:nlsmCherry)*^*ia49*^ line the mRNAs coding for a constitutively active form of YAP1, TAZ or TEAD (YAP-5SA, TAZ-4SA, and TEAD-VP16 respectively). As expected, the injection of any of these mRNAs significantly increased *Hsa*.*CTGF*:nlsmCherry expression (Fig. 2B–D). A similar responsiveness of the reporter signal upon Yap1/Taz activity modulation was found for *Tg(Hsa*.*CTGF:eGFP)*^*ia48*^ (data not shown). We also validated more specifically that the reporter signal is dependent on Yap/Taz in a number of different tissues, such as the eye, the heart, the floorplate, and the major axial vessels (Fig. S3).

We further tested the *Hsa*.*CTGF*:nlsmCherry transgene by treating the reporter embryos with Dexamethasone (DEX), a synthetic glucocorticoid recently shown to promote the nuclear activity of YAP1 *in vitro*^39^. 24 hpf *Tg(Hsa*.*CTGF:nlsmCherry)*^*ia49*^ embryos were exposed to a solution containing 25 μM of DEX and analyzed after 24 h. DEX treated embryos displayed a significant increase of fluorescence when compared with controls, confirming *in-vivo* that glucocorticoids activate Yap1/Taz/Tead mediated transcription. Interestingly, we also observed that DEX treatment does not increase the reporter signal in Yap1/Taz knockdown embryos, thus confirming that the response induced by glucocorticoids is mediated by Yap/Taz activation. (Fig. S4A-A”).

Recently, it has been shown that blood flow can induce nuclear import of YAP1 and its transcriptional activity^18^. We thus tested *Hsa*.*CTGF* zebrafish reporter with *silent heart* (*sih/tnnt2a*) morpholino to block cardiac contractility and blood flow; *sih* morphants showed a significant reduction of *mCherry* transgene expression in endothelial cells when compared to control morpholino-injected embryos (Fig. S4B).

Taken together, these experiments demonstrate that the *Tg(Hsa*.*CTGF:nlsmCherry)*^*ia49*^ and *Tg(Hsa*.*CTGF:eGFP)*^*ia48*^ lines can be used to analyze Yap1/Taz activity *in vivo*.

### *Tg(Hsa*.*CTGF:nlsmCherry)*^*ia49*^ zebrafish reveals *in vivo* the spatio-temporal activation of Yap1/Taz

We next sought to identify the embryonic stages and tissues in which the reporter is active. Due to maternal expression, fluorescence is ubiquitously detectable at the dome stage and during epiboly in embryos derived from *Tg(Hsa*.*CTGF:nlsmCherry)*^*ia49*^ females crossed to wild-type males (Fig. S5A). Consistently, mCherry expression is prominent in the adult ovary (the organ with the strongest reporter signal in adults), where the mCherry protein is clearly visible in the nuclei of the oocytes and the accompanying follicle cells (Fig. S5B,C).

**Figure 3:**
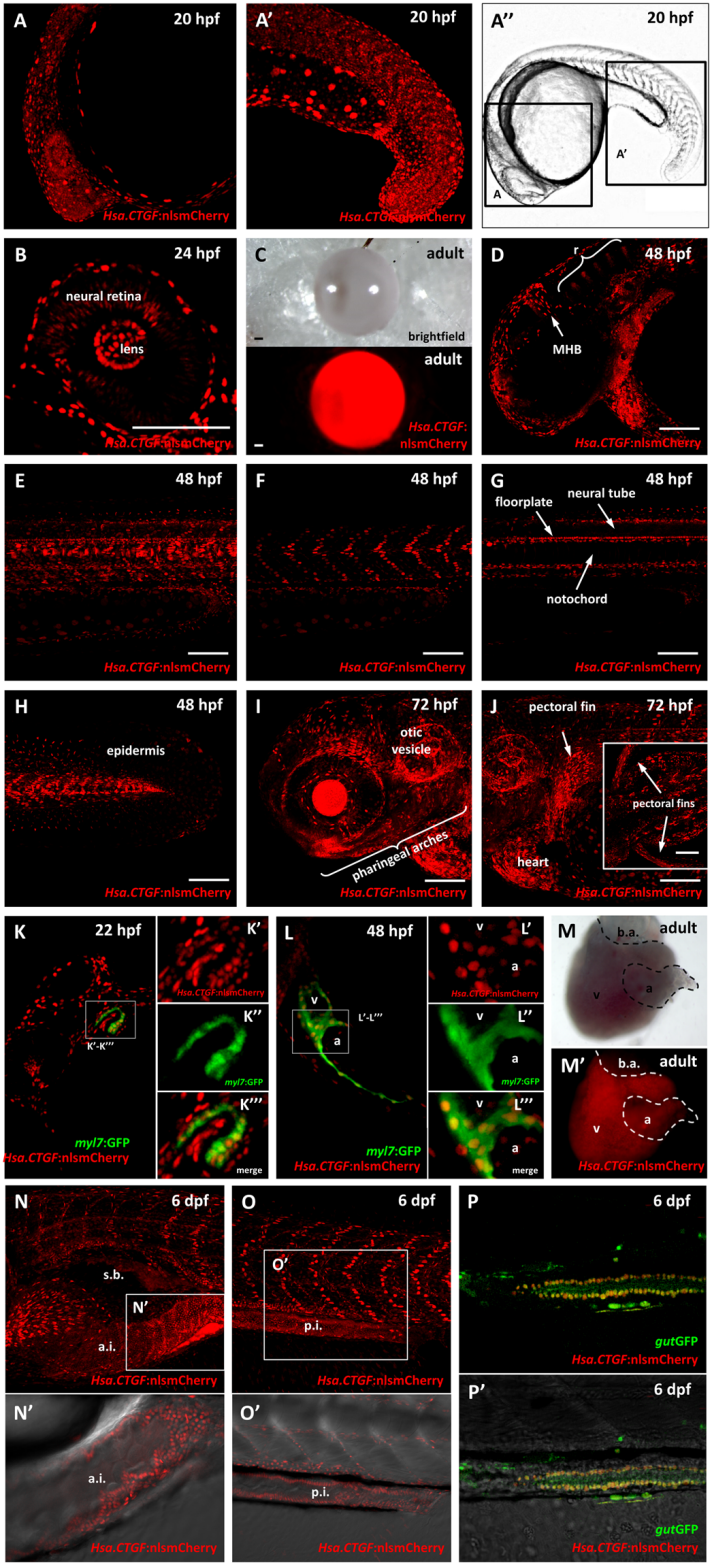
Analysis of *Tg(Hsa.CTGF:nlsmCherry)*^*ia49*^ reporter activation. (**A**–**A**”) *In vivo* confocal images of *Hsa*.*CTGF*:nlsmCherry fluorescence at 20 hpf. (**B**) Confocal sagittal section of the eye at 24 hpf. (**C**) Lens of a *Tg(Hsa*.*CTGF:nlsmCherry)*^*ia49*^ adult fish. (**D**) Confocal Z-stack projection at 48 hpf showing the transgene expression in the rhombencephalon (r) and the midbrain-hindbrain boundary (MHB) regions. (**E**) Confocal Z-stack projection of the trunk at 48 hpf. (**F**–**G**) Sections of the Z-stack projection in (**E**) are shown to highlight reporter activation in the somatic muscle (**F**), in the floorplate, the neural tube and the notochord (**G**). A strong signal is detected in a row of nuclei located between the neural tube and the notochord and representing the floorplate. The neural tube is only weakly positive. Below the floorplate, the notochord is expressing mCherry in the nuclei located just dorsally and ventrally with respect to the dark stripe of the notochord cells vacuoles. (**H**) Reporter fluorescence is visible in the epidermis along the whole embryo; here in a confocal Z-stack projection of the terminal portion of the tail at 48 hpf. (**I**–**J**) Confocal Z-stack projections of the rostral region of a 72 hpf reporter embryo. In the inset, a dorsal view highlighting the mCherry expression in the pectoral fins is depicted. (**K**) Confocal sagittal section of the heart region of a *Tg(Hsa*.*CTGF:nlsmCherry)*^*ia49*^*/Tg(myl7:GFP)* double transgenic embryo at 22 hpf. (**K**’–**K**”’) Inset of K: mCherry channel (**K’**), GFP channel (**K”**), merge (**K”’**). (**L**) Confocal sagittal section of the heart at 48 hpf. (**L’**–**L”’**) Inset of L: mCherry channel (**L’**), GFP channel (**L”**), merge (**L”’**). (**M**–**M’**) Heart of a *Tg(Hsa*.*CTGF:nlsmCherry)*^*ia49*^ adult fish. (**N**,**O**) Confocal Z-stack projections at 6 days post fertilization (dpf), showing the strong transgene expression in the whole intestine. (**N’**) Inset of N, single confocal sagittal section, bright field and mCherry merge, focusing on the reporter expression in the anterior intestine. (**O’**) Inset of O, single confocal sagittal section, bright field and mCherry merge, focusing on the reporter expression in the mid-posterior intestine. (**P**) Confocal sagittal section of the mid-posterior intestine of a 6 dpf *Tg(Hsa*.*CTGF:nlsmCherry)*^*ia49*^*/Tg(gut:GFP)*^*s854*^ double transgenic larva. (**P’**) Merge with bright field. r: rhombencephalon; MHB: midbrain-hindbrain boundary; v- ventricle; a: atrium; b.a.: bulbus arteriosus; s.b.:swim bladder; a.i.: anterior intestine; p.i.: posterior intestine. Scale bar: 100 μm.

The first zygotic expression of the reporter protein in transgenic embryos (derived from *Tg(Hsa*.*CTGF:nlsmCherry)*^*ia49*^ males crossed to wild-type females) is detectable at late somitogenesis. At 20 hpf fluorescence is found widely across the developing embryo, with the strongest signal localized in the mesenchyme of the tail bud (Fig. 3A). By 24 hpf, fluorescence is observed in many tissues and organs, such as eyes, heart, midbrain-hindbrain boundary (MHB), rhombencephalon, epidermis, muscles, neural tube, notochord, floorplate and vasculature. Reporter expression is persistent in those districts even during later developmental stages (Fig. 3B–H). In the eye the signal is strong in the lens and is also present in the neural retina (Fig. 3B). The lens remains strongly fluorescent until adulthood (Fig. 3C). In the dorsal portion of the head, two regions display transgene activation by 24 hpf: the MHB and the rhombencephalon. In the rhombencephalic region *Hsa*.*CTGF*:nlsmCherry fluorescence is present in six stripes of cells that follow the metameric organization of rhombomeres (Fig. 3D). At 72 hpf, besides the lens, the reporter signal is particularly strong in the pharyngeal arches (mainly in the mandibular one), otic vesicles, pectoral fins and heart (Fig. 3I,J). A time series of the reporter expression during embryonic and early larval stage is shown in Fig. S6.

In the heart, fluorescence is already visible at early stages and persists throughout adulthood (Fig. 3K–M). To confirm the expression in the cardiac progenitors and specifically in the cardiomyocytes, we outcrossed the *Tg(Hsa*.*CTGF:nlsmCherry)*^*ia49*^ line to the *Tg(myl7:GFP)* line^40^. As indicated by the co-localization between mCherry and eGFP from 22 hpf, the Yap1/Taz reporter is active in cardiac progenitors as well as in differentiated cardiomyocytes (Fig. 3K,L), in agreement with previously described zebrafish reporter lines^41,42^ and with genetic data in mammals, where YAP1 is active together with TEADs to promote the growth of embryonic and fetal cardiomyocytes^11,43–46^. *Hsa*.*CTGF:*nlsmCherry fluorescence is visible also in the intestinal epithelium along the whole intestine (Fig. 3N–P). Specific expression in the epithelium of the intestine is demonstrated by fluorescence co-localization at cellular resolution in *Tg(Hsa*.*CTGF:nlsmCherry)*^*ia49*^*/Tg(gut:GFP)*^*s854*^ ^47^ double transgenic embryos (Fig. 3P).

**Figure 4:**
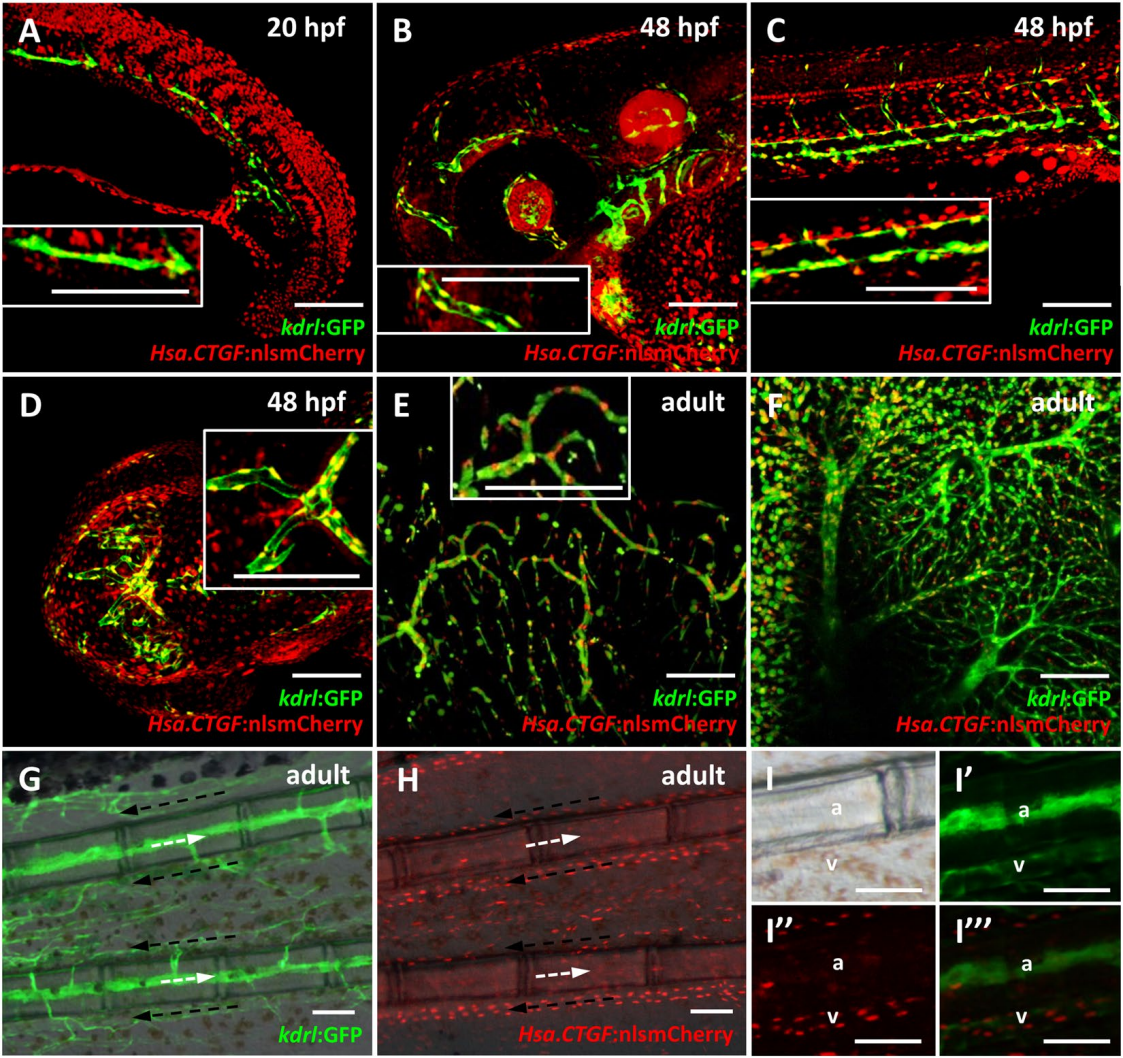
*Tg(Hsa*.*CTGF:nlsmCherry)*^*ia49*^ reporter activity is prominent in the endothelium. (**A**–**D**) Reporter expression in the endothelium during development from 20 to 48 hpf. (**E**–**I**) Reporter expression in the adult endothelium. (**A**) Confocal sagittal section of the trunk of a 20 hpf *Tg(Hsa*.*CTGF:nlsmCherry)*^*ia49*^*/Tg(kdrl:GFP)* double transgenic embryo. *Tg(kdrl:GFP)* expresses GFP in all endothelial cells. Yap1/Taz are active in the endothelial cells in the developing vessels, as shown by the co-localization between the two signals. (**B**–**D**) Confocal Z-stack projections of the head region (side view in (**B**), dorsal view in (**D**)) and the trunk (**C**) at 48 hpf. Reporter signal co-localizes with *kdrl*:GFP expression throughout the embryo. (**E**,**F**) Confocal Z-stack projection of brain (**E**) and liver (**F**) tissue of a double transgenic adult fish, displaying Yap1/Taz reporter activation in the endothelium of respectively the cerebral and hepatic vascular networks. The insets represent zoomed views highlighting the co-localization between *Hsa*.*CTGF*:nlsmCherry and *kdrl*:GFP. (**G**–**I**) Fluorescent microscope images of adult caudal fin in *Tg(kdrl:GFP)* (**G**) and *Tg(Hsa*.*CTGF:nlsmCherry)*^*ia49*^ (**H**). Lateral views, anterior to the left, dorsal to the top. Arterial (white arrows) and venous (black arrows) bloodstream is indicated. Yap1/Taz reporter activity is stronger in the veins running laterally to the bony fin rays with respect to arteries inside the bony rays, as highlighted in the single channels and merge magnifications (**I**–I”’). a: artery; v-vein. Scale bar: 100 μm.

Finally, one prominent site of *Hsa*.*CTGF:nlsmCherry* expression is the vascular network, where the reporter signal is visible as soon as the vasculature starts to develop and preserved during adulthood. The co-localization of *Tg(Hsa*.*CTGF:nlsmCherry)*^*ia49*^/*Tg(kdrl:GFP)* double transgenics indicates specific activation of Yap1/Taz in endothelial cells throughout the 20 hpf and 48 hpf embryos, as well as in adult fish (Fig. 4A–I).

### Yap1/Taz are required for posterior cardinal vein development

To study the role played by Yap1/Taz in zebrafish development, and more specifically in the vasculature, *yap1* and *taz* mutants (*yap1*^*bns19*^ and *taz*^*bns35*^, respectively) generated by CRISPR/CAS9 were used^48,49^. The *yap1*^*bns19*^ allele contains a 41 bp deletion in exon 1 of *yap1*, while the *taz*^*bns35*^ allele contains a 29 bp insertion in exon 2 of *taz*. Both frame-shift mutations are predicted to encode a truncated protein(Fig. S7A,B). The reduction of Yap1/Taz activity in the mutants was confirmed by the analysis of the *Tg(Hsa*.*CTGF:nlsmCherry)*^*ia49*^ reporter line. As expected, we observed a decrease of *Hsa*.*CTGF:*nlsmCherry expression in the endothelium of 48 hpf *yap1*^*bns19*^ and *taz*^*bns35*^ mutant embryos (Fig. S7C,C’).

While we did not observe striking vascular phenotypes in *taz* mutants, the primary vascular phenotypes of *yap1* mutants have been described recently^18^. Here, we report additional alterations in the cranial and ocular vasculature: the mesencephalic vein (MsV) and the dorsal longitudinal vein (DLV) were truncated in more than 50% (5/9) of *yap1*^−/−^ larvae analyzed at 72 hpf; at 5 dpf, fewer hyaloid vessels could be observed in the eyes (Fig. S8), a number further reduced in colobomatous eyes.

**Figure 5:**
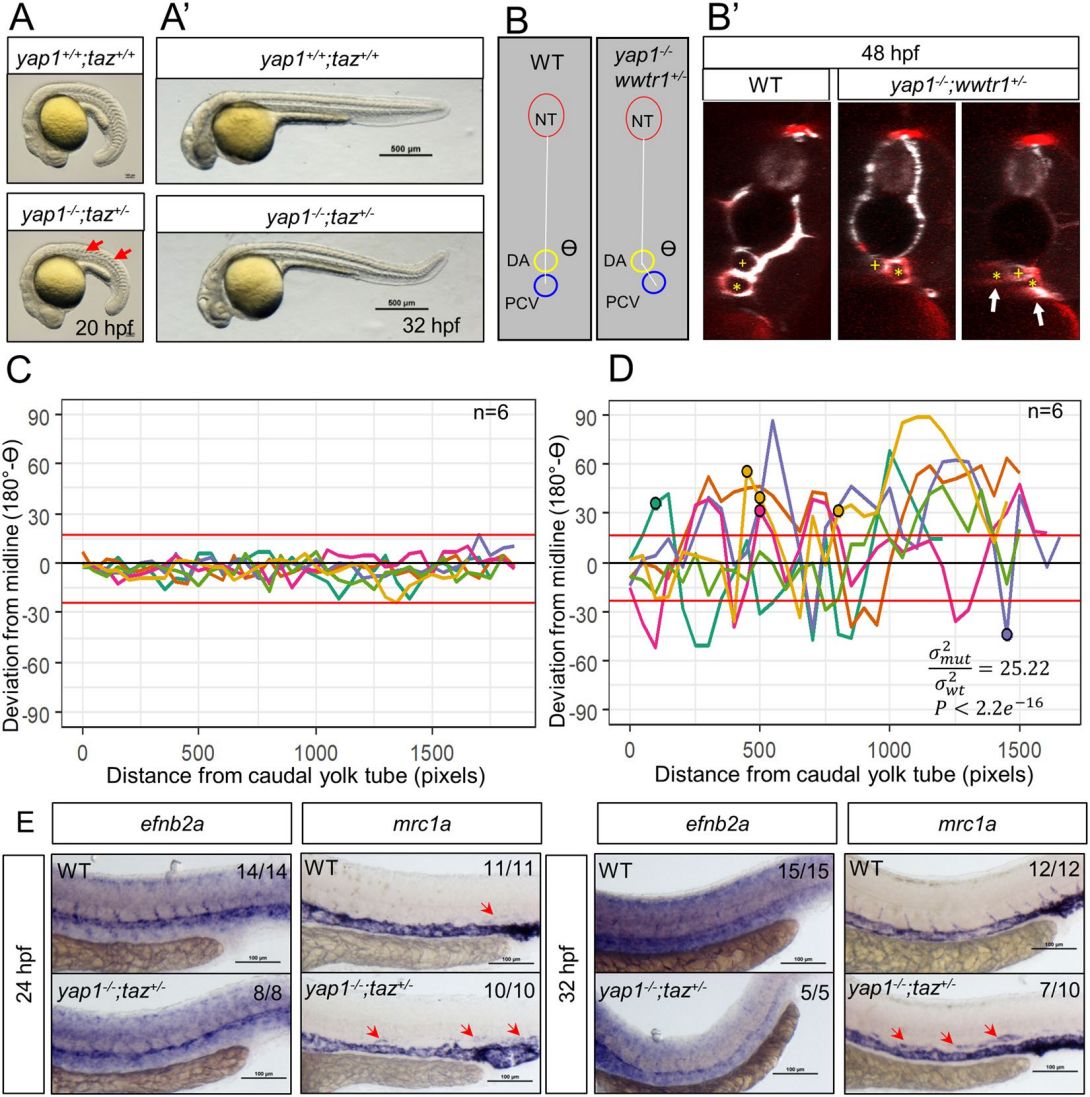
Yap1/Taz are required for PCV development. (**A**) Gross morphology of 20 hpf WT and *yap1*^−/−^*;taz*^+*/*−^ embryos. Red arrows point to the undulating notochord. (**A’**) Gross morphology of 32 hpf WT and *yap1*^−/−^*;taz*^+*/*−^ embryos. (**B**,**B’**) Transverse sections of the trunk revealing the relative positions of the neural tube (NT), dorsal aorta (DA) and posterior cardinal vein (PCV). The PCV of *yap1*^−/−^*;taz*^+*/*−^ animals has deviated from the midline and appears to split into two lumenized vessels (arrows). White signal, *etv2:*EGFP transgene expression; red signal, *lyve1b*:DsRed transgene expression. The PVC lumen is marked with an asterisk (*), while the DA lumen is marked with a yellow “ + ”. (**C**,**D**) Analysis of the deviation of the PCV from the midline in 48 hpf embryos. The data are obtained from a series of transverse sections starting from the caudal end of the yolk tube and moving 50 sections rostrally. The NT, DA and PCV were manually demarcated and the deviation of the PCV from the midline was defined as β = 180°-ϴ. With ϴ (see panel **B**) we defined the angle formed by the NT, DA, and PCV in each transverse section. Each value of β was plotted for WT and *yap1*^−/−^*;taz*^+*/*−^ trunks at 48 hpf. Each line represents a single animal. 4/6 of *yap1*^−/−^*;taz*^+*/*−^ embryos exhibited a PCV that appears to split into two lumenized vessels that are *lyve1b*:DsRed positive (marked with a circle on the graph). Red horizontal lines above and below 0° are the WT maxima and minima. *P* values were calculated by the F-test, which tests whether the spread of angles (180° - ϴ) between WT and mutants is the same. σ_mut_^2^: variance of (180° - ϴ) in *yap1*^−/−^*;taz*^+*/*−^ animals; σ_WT_^2^: variance of (180° - ϴ) in WT animals. (**E**) Whole mount *in situ* hybridization (WISH) for the expression of *efnb2a* and *mrc1a*, markers of arteries and veins, respectively. Red arrows point to expression of *mrc1a* in the region of the DA. The fraction of the embryos exhibiting the phenotype shown in each image was reported in the upper right corner of the corresponding panel. Scale bars, 100 μm.

Double *yap1;taz* homozygous mutants exhibit severe developmental defects and die by 30 hpf as previously reported^18,48,50^. Therefore, we chose to analyze the animals retaining one allele of *taz*. *yap1*^−/−^*;taz*^+*/*−^ embryos exhibited an undulating notochord at 20 hpf and curving of the tail by 32 hpf (Fig. 5A,A’). Moreover, about 16% (7/45) of *yap1*^−/−^*;taz*^+*/*−^ embryos had bifid hearts and 76% (34/45) did not exhibit blood circulation; the remaining 9% (4/45) of mutants exhibited blood circulation, but not at WT levels.

In addition, we observed an intriguing phenotype at the level of the axial vessels in the *yap1*^−/−^*;taz*^+*/*−^animals. At 30 hpf, when analyzed in transverse confocal sections, embryos were found to exhibit a posterior cardinal vein (PCV) deviating from the midline (Fig. S9). This phenotype worsened and was fully penetrant at 48 and 72 hpf (Figs 5B–D and S10). Interestingly, in 67% (4/6) of 48 hpf *yap*^−/−^*;taz*^+*/*−^embryos the PCV appeared to exhibit two distinct lumens (Fig. 5B).

We aimed to investigate whether these PCV alterations might be associated with defects of arterial/venous specification during axial vessel development. *In situ* hybridization for expression of the arterial marker *efnb2a* showed that it was not affected in *yap1*^−/−^*;taz*^+*/*−^ embryos at 24 and 32 hpf. On the contrary, expression of *mrc1a* (a marker for veins and lymphatics^51^) was altered in *yap1*^−/−^*;taz*^+*/*−^ embryos when compared to WT siblings (Fig. 5E). During development, *mrc1a* is initially expressed in the presumptive venous progenitors localized in the axial vessel, becoming restricted to the PCV and other veins as well as in lymphatic vessels^51^. In 70% (7/10) of 32 hpf *yap1*^−/−^*;taz*^+*/*−^ embryos, some *mrc1a* expression was observed in the DA, unlike in WT animals (Fig. 5E), suggesting a defect in DA specification.

Taken together, these findings confirm the role of Yap1/Taz in vascular development and reveal its specific requirement for the cranial and ocular vascular networks, as well as for the specification and organization of the axial vessels.

### The upregulation of Yap1/Taz/Tead-mediated transcription causes aberrant vessel sprouting

**Figure 6:**
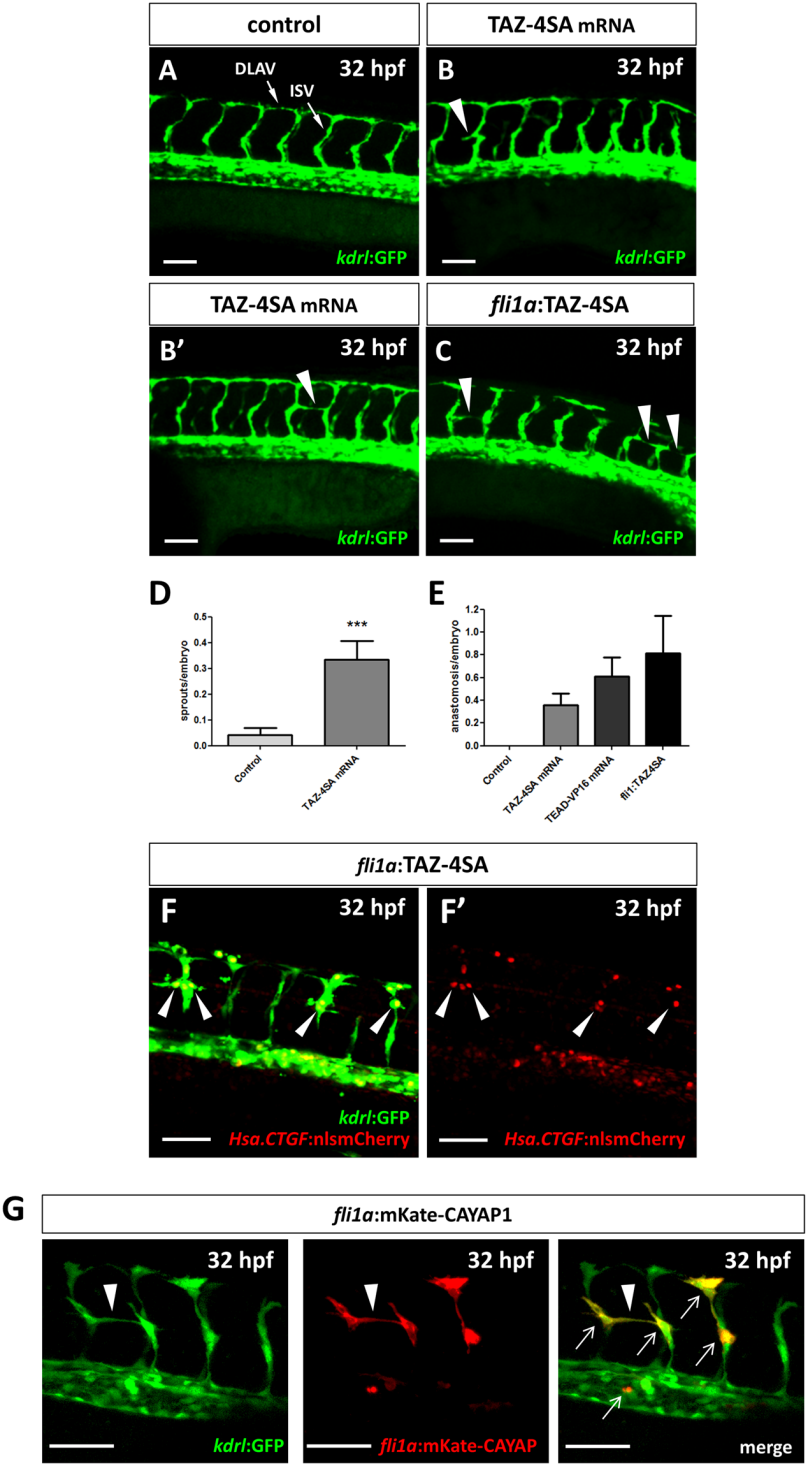
Yap1/Taz activity upregulation promotes vessel sprouting. (**A**–**C**) Confocal Z-stack projections of the midtrunk region of *Tg(kdrl:GFP)* 32 hpf embryos. (**A**) Representative image of a control injected embryo. (**B**,**B’**) Two TAZ-4SA mRNA injected embryos, showing an aberrant ISV sprout (arrowhead in **B**) and an anastomosis between adjacent ISVs (arrowhead in **B’**). (**C**) A mosaic embryo injected with pDestTol2CG2-fli1a-TAZ-4SA plasmid, showing three anastomosis between adjacent ISVs (arrowheads in **C**). (**D**,**E**) Quantification of the aberrant sprouting caused by Yap1/Taz activity upregulation. The number of non-anastomosed aberrant sprouts (**D**) and the number of anastomosis (**E**) between adjacent ISVs were evaluated. Both phenomena, observed in TAZ-4SA mRNA and pDestTol2CG2-fli1a-TAZ-4SA mosaic injected embryos, are extremely rare or absent at all in the controls. Controls: n = 49; TAZ-4SA mRNA-injected: n = 42; TEAD-VP16 mRNA-injected: n = 31; pDestTol2CG2-fli1a-TAZ-4SA plasmid-injected: n = 16. (**F**,**F’**) *Tg(Hsa*.*CTGF:nlsmCherry)*^*ia49*^*/Tg(kdrl:GFP)* double transgenic embryos injected with the pDestTol2CG2-fli1a:TAZ-4SA vector. A strong overactivation of the *Hsa*.*CTGF* reporter signal was observed in the nuclei of the endothelial cells undergoing anomalous sprouting (arrowheads in **F**) with respect to the other normal ISVs. (**G**) In mosaic embryos injected with the PCS2-fli1a:CAYAP-mKate plasmid a specific expression of the mKate was reported in conjunction with the anomalous endothelial sprouts (arrowhead). The plasmid is endothelium-specific, as highlighted by the co-localization (arrows) between the mosaic mKate and the GFP of the stable transgenic line *Tg(kdrl:EGFP)*. Lateral view, anterior to the left, dorsal to the top. ***p < 0.001. ISV: intersegmental vessel; DLAV: dorsal longitudinal anastomotic vessel. Scale bar: 50 µm.

The vascular developmental defects observed in *yap1;taz* mutants and the emerging role of Yap1/Taz in developmental angiogenesis^16–18^, prompted us to explore the effect of the upregulation of Yap1/Taz activity on embryonic angiogenesis in zebrafish. Therefore, we injected a constitutively active form of Taz (TAZ-4SA mRNA) in one-cell stage *Tg(kdrl:GFP)*^*s843*^ embryos. While no alteration was observed in PCV development, at 32 hpf we observed the appearance of abnormal sprouts emerging horizontally from some ISVs in TAZ-4SA injected embryos (Fig. 6A,B–D). These abnormal sprouts pointed mostly toward the adjacent ISVs, and often gave rise to complete anastomosis between two adjacent ISVs (Fig. 6B’). Despite the low frequency of aberrant vessel sprouting in TAZ-4SA injected embryos, this phenomenon was never observed in control fish. In particular, complete anastomosis between adjacent ISVs was never detected in more than 50 injected control embryos analysed (Fig. 6A,B’,E). To further confirm the aberrant sprouting phenotype, the experiment was repeated using YAP-5SA mRNA, obtaining similar results (data not shown).

We next asked whether the effect on ISVs angiogenesis was due to enhanced Yap1/Taz/Tead-mediated transcription, or to other non-transcriptional functions of Yap1 and Taz proteins. To answer this question, *Tg(kdrl:GFP)*^*s843*^ embryos were injected with TEAD-VP16 mRNA, which constitutively activates Tead target gene transcription independently of Yap1/Taz. Aberrant vessel sprouting and anastomosis between ISVs were found also after the injection of TEAD-VP16 mRNA, thus confirming the vessel sprouting-promoting ability of Yap1/Taz/Tead-mediated transcription (Fig. 6E).

To address whether the effects of Yap1 and Taz on ISVs was specifically due to their activity in endothelial cells, we designed a new construct, placing TAZ-4SA expression under the control of the *fli1a* endothelial-specific promoter. To perform this experiment, we used the pDestTol2CG2 transposon backbone, containing the cardiac *myl7*:GFP transgenesis marker, in order to detect the mosaicism levels of injected fish^52^. We speculated that with these experimental conditions we would have been able to detect the presence of aberrant sprouting phenotype only if the vessel sprouting-promoting ability of Yap1/Taz had been due to its transcriptional activity in endothelial cells. After the injection of pDestTol2CG2-fli1a-TAZ-4SA plasmid in one-cell stage *Tg(kdrl:GFP)* embryos, we observed that about 44% (7/16) of the mosaic embryos bearing endothelial-specific TAZ overexpression exhibited the aberrant vessel sprouting effect induced by YAP-5SA or TAZ-4SA mRNAs injection (Fig. 6C,E). Moreover, by injecting pDestTol2CG2-fli1a-TAZ-4SA plasmid in *Tg(Hsa*.*CTGF:nlsmCherry)*^*ia49*^*; Tg(kdrl:GFP)*^*s843*^ embryos, we detected a strong induction of *Hsa*.*CTGF*:nlsmCherry reporter expression (i.e. Yap1/Taz nuclear activity) specifically in endothelial cells undergoing sprouting (Fig. 6F). These data indicate that the upregulation of Yap1/Taz activity in single endothelial cells is sufficient to promote aberrant ISVs sprouting.

We further confirmed the autonomous nature of the phenotype and ruled out the possibility that mosaic expression outside of the promoter-specific domains upon DNA injection might be partially responsible for the observed sprouting. We carried out an endothelium-specific Yap1/Taz upregulation experiment by transiently expressing an activated form of *Yap1* (*yap1-1β*^*S87A*^ or CAYAP1)^53^ fused with the fluorescent protein mKate. The injection of the PCS2-fli1a-mKate-CAYAP1 plasmid in one-cell stage *Tg(kdrl:GFP)* embryos allowed us to detect, with one-cell resolution, the vessel domains in which we were actually upregulating the pathway and to demonstrate the correlation between vascular abnormalities and vascular-specific CAYAP1 expression. Similar to the pDestTol2CG2-fli1a:TAZ-4SA system, in the mosaic PCS2-fli1a-mKate-CAYAP1 injected embryos we observed a specific expression of the mKate-CAYAP1 in conjunction with the ISVs undergoing anomalous endothelial sprouting (Fig. 6G).

## Discussion

YAP1/TAZ signaling has recently gained much attention in developmental, cancer and regeneration biology. However, most data in adult mammalian tissues indicate that YAP1/TAZ are dispensable for homeostatic self-renewal and become required only upon genetic deletion of inhibitory cues such as Hippo, or upon inflammatory and tumorigenic stimuli; this led to the still unresolved issue of whether YAP1/TAZ are active or not in most adult tissues. Here, we described the generation, validation and characterization of a Yap1/Taz zebrafish reporter, which represents a powerful tool to follow this signaling activity in a living vertebrate, offering also interesting applications in drug screening, cancer and regenerative biology.

Validation through knockdown and overexpression approaches demonstrated that the *Hsa*.*CTGF*-based zebrafish transgenic lines faithfully report *in vivo* Yap1/Taz activity, being able to reveal significant corresponding variations. A first Yap1/Taz zebrafish reporter, named *4xGTIIC*, has been previously published by Miesfeld and Link^41^. During embryogenesis, a strong Yap1/Taz activity was observed in the same regions of the *Hsa*.*CTGF*-based reporter presented here, such as the epidermis, heart, otic and lens vesicles, midbrain-hindbrain boundary (MHB) region and striated muscle of the trunk. However, we described a much wider expression pattern for the *Hsa*.*CTGF* reporter with respect to that described for the *4xGTIIC*, although the latter was mainly described with a destabilized GFP-expressing line, which makes hard to do comparisons. The *Hsa*.*CTGF* transgenic line allowed us to point out regions of Yap1/Taz nuclear activity previously not described in the other reporters for the pathway (rhombencephalon, neural tube, notochord, floorplate, pharyngeal arches, and pectoral fin). The *Hsa*.*CTGF* reporter activation in the myl7 positive cardiac precursor cells and in the heart is consistent with that of the *Tg(eef1a1l1:Gal4db-TEAD2ΔN-2A-mCherry);(UAS:GFP)* reporter developed by Fukui and colleagues^42^. The activity in the endothelium, not shown in the *4xGTIIC* reporter, is also consistent with the *Tg(eef1a1l1:Gal4db-TEAD2ΔN-2A-mCherry);(UAS:GFP)* reporter^18^, as well as with the endothelium-specific reporter described by Nagasawa-Masuda and Terai^20^. Yap1 and TEAD2 transcriptional activity has been shown to be modulated *in vivo* by blood flow^18^ and, similarly, we confirmed the positive regulation exerted by the circulation on Yap1/Taz activity by showing that the also the expression of *Hsa*.*CTGF*:nlsmCherry in endothelial cells is modulated by blood flow. Notably, the *Hsa*.*CTGF* reporter is responsive to the synthetic glucocorticoid Dexamethasone, confirming *in vivo* the recent findings of Sorrentino and colleagues^39^, and highlighting the potential application of *Hsa*.*CTGF* reporter lines in drugs screenings.

A potential criticism might be that the *Hsa*.*CTGF* Yap1/Taz transgenic lines are reporting the expression pattern of the zebrafish Yap1/Taz target genes *ctgfa* rather than the global TEAD-dependent Yap1/Taz signaling activity. There are several reasons ruling out this hypothesis: (i) the reporter expression is driven by a 200 bp fragment of the human *CTGF* promoter, that represents only a minimal part with respect to the whole promoter regulating the expression of the *CTGF* gene. Pfefferli and Jazwinska analyzed all the transcription factor binding sites within the 3.18 kb upstream regulatory sequence of *ctgfa*, showing that the vast majority of binding sites are outside the 200 bp fragment that was used to drive the *Hsa*.*CTGF* transgene expression^54^. (ii) The zebrafish *Tg(ctgfa:EGFP)* reporter based on the 3.18 kb *ctgfa* promoter described by Pfefferli and Jazwinska showed a different expression pattern from the *Hsa*.*CTGF* reporter, being limited to the notochord, the heart and the connective tissue of regenerating fins^54,55^. (iii) In spite of a partial overlap (lens, otic vesicles, heart, pharyngeal arches, pectoral fin, and floorplate), the expression of the *ctgfa/b* gene and the *Hsa*.*CTGF*:nlsmCherry transgene is different. For instance, *in situ* hybridization performed to detect the mCherry expression on 48 and 72 hpf *Tg(Hsa*.*CTGF:nlsmCherry)* reporter embryos (data not shown) clearly labels the MHB and the rhombencephalic regions, which are not positive for *ctgfa/b* expression^56^. On the contrary, ctgfa is expressed in the pancreatic bud (https://zfin.org/ZDB-FIG-060130-1737) while the *Hsa*.*CTGF* reporter doesn’t exhibit transgene activity in this region.

The analysis of the spatio-temporal activation of *Hsa*.*CTGF* reporter revealed a wide activation of Yap1/Taz signaling during early embryonic development, with a stronger signal in the proliferating and undifferentiated tail bud mesenchyme. This is likely reflecting the main function for YAP1/TAZ as transducers of the Hippo pathway: promotion of cell proliferation and organ growth during development^44,57,58^. In adulthood, YAP1/TAZ expression is strongly limited, being enriched in the stem/progenitor cells niches^59^. Consistently, the *Hsa*.*CTGF* reporter is largely silenced in the fully-grown fish with respect to the embryonic and larval development. Highly positive organs or tissues in the adult include the ovary, the lens, the heart and the endothelium. While in the lens the presence of the reporter protein could be simply due to almost absent protein turnover of these cells^60^, the persistence of Yap1/Taz activity in the cardiovascular system suggests its important role in the maintenance of cardiac and vascular functions. This is in agreement with the requirement of Yap1 for the maintenance of blood vessels during zebrafish development^18^.

*yap1* and *taz* single knockouts, unlike the double mutants, do not exhibit significant vascular phenotypes, implying a functional redundancy of the two genes during vascular development. Nevertheless, we described slight defects in the cranial and ocular vasculatures of the *yap1*^−/−^ embryos, phenotypes not reported in previous works. While the specific reduction of the number of hyaloid vessels in *yap1* mutants might be a consequence of coloboma, *yap1*^−/−^*;taz*^+*/*−^ animals exhibited an unusual phenotype during the formation of the axial vessels: the PCV deviates intermittently from the midline, and occasionally exhibits two distinct lumens. Analysis of arterial/venous-specific markers revealed that defective specification of the DA is also observed. Specifically, the expression of *mrc1a* in 32 hpf *yap1*^−/−^*;taz*^+*/*−^ embryos is not restricted to the PCV and venous/lymphatic vessels as in WT^51^, but is still observed in the DA.

Notably, the overactivation of Yap1/Taz in the endothelium was sufficient to cause an abnormal vessel sprouting phenotype with formation of atypical anastomosis between adjacent ISVs, a result consistent with recent evidences *in vitro* and in mouse models on the role of YAP1/TAZ in angiogenesis^16,17,61–63^.

Altogether, our results present a novel comprehensive *in vivo* view of Yap1/Taz activity during development and adulthood at the whole organism level. Together with the vascular phenotypes displayed by *yap1/taz* mutants and upon endothelium-specific upregulation of Yap1/Taz/Tead-mediated transcription, it confirms and further extends the emerging role of Yap1/Taz signaling in vessel maintenance and developmental angiogenesis.

## Material and Methods

### Animals

All live animal procedures were approved by the institutional ethics committee for animal testing of the University of Padua and the Max Planck Society as well as in accordance with the relevant guidelines and regulations of Italy, Germany and European Union.

To inhibit pigment formation, embryos and larvae were incubated in 0.003% 1-phenyl-2-thiourea (PTU). The following fish lines were used and outcrossed either to wild-type fish or to the *Tg(Hsa*.*CTGF:nlsmCherry)*^*ia49*^ Yap1/Taz reporter line: *Tg(myl7:GFP)*^64^, *Tg(Xla*.*Eef1a1:GFP)*^*s854* ^^47^ (gut/GFP), and *Tg(kdrl:GFP)*^*s843* ^^65^ *Tg(−5*.*2lyve1b:dsRed)*^*nz101*^ ^66^, *TgBAC(etv2:EGFP)*^*ci1*^ ^67^, *Tg(fli1a:EGFP)*^*y1*^ ^68^, and *Tg(2xID1BRE:nlsmCherry)*^*ia17*^ ^69^. In the *TgBAC(etv2:EGFP)*^*ci1*^ line, the GFP marks the whole vascular system under the promoter control of the early regulator of vascular and myeloid development *etsrp/etv2*. In *Tg(−5*.*2lyve1b:DsRed)*^*nz101*^ line the DsRed is expressed in major axial veins and the lymphatic system under the control of the *lyve1* promoter. Mutant alleles used in this paper are: *yap1*^*bns19* ^^48^ and *taz*^*bns35* ^^48,49^.

### Generation of *Tg(Hsa.CTGF:eGFP)*^*ia48*^ and *Tg(Hsa.CTGF:nlsmCherry)*^*ia49*^

The −200/+ 27-*CTGF* promoter fragment was amplified by PCR from a human genomic DNA using the following oligonucleotides:

Hsa.CTGF-for (5′-TCTAGAAGATCTTCTGTGAGCTGGAGTGTGC-3′) and Hsa-CTGF-rev (5′-AAGCTTCCATGGAGCGGGGAAGAGTTGTTGT-3′). The *CTGF* promoter fragment was subcloned in the Gateway 5′ entry vector pME-MCS (Invitrogen) using the BglII and HindIII restriction enzymes. The resulting p5E-Hsa.CTGF entry vector was recombined with the nlsmCherry, and eGFP-carrying middle entry vectors and the p3E-polyA entry clone containing the SV40 late polyA signal (Invitrogen). Entry plasmids were recombined into the Tol2 destination vector pDestTol2pA2 (Invitrogen) through a MultiSite Gateway LR recombination reaction as previously described^52^. 30 pg of Tol2 destination vectors and 25 pg of Tol2 transposase mRNA^70^ were co-injected into one cell-stage wild-type zebrafish embryos. Microinjected embryos were selected for mosaic transgenic expression at 24 and 72 hpf using an epifluorescent microscope, raised to adulthood and outcrossed to wild-type fish. Overall, 13 out of 17 screened fish were identified as founders. Founder fish for each reporter line, named *Tg(Hsa*.*CTGF:eGFP)*^*ia48*^ and *Tg(Hsa*.*CTGF:nlsmCherry)*^*ia49*^, were selected for fluorescence level and the number of transgenic insertions in order to establish stable transgenic lines with a single insertion.

### Morpholinos, mRNA and plasmids injections

The antisense morpholino oligos were obtained from Gene Tools, LLV (U.S.). The following splice blocking (MO-Yap and MO-Taz) and control morpholinos were used: MO-*Yap1*: 5′-GCA ACA TTA ACA ACT CAC TTT AGG A-3′^71^; MO-*Taz*: 5′-GTA TGT GTT TCA CAC TCA CCC AGG T-3′; MO-*tnnt2a (sih)*: 5′-CAT GTT TGC TCT GAT CTG ACA CGC A-3′^72^ MO-ctrl: 5′-AGA ACA TAA TCA GTA GTG TTC GA-3′. The MO stock solution (1 mM) was diluted in Danieau’s solution, and ∼1 nL was injected per embryo as previously described^73^.

YAP-5SA is a constitutively active version of the human YAP1 protein, which has been mutated in its five key serine residues, resulting insensitive to LATS1/2-dependent phosphorylation and sequestration in the cytoplasm. Analogously, TAZ-4SA is the constitutively active version of the murine TAZ protein, mutated in its four serine residues recognized by LATS1/2. TEAD-VP16 is a fusion protein of the N-terminal region of TEAD transcription factor and the activation domain of herpes simplex virus VP16. TEAD-VP16 does not need any transcriptional co-activator to work, leading again to a constitutive transcription of its target genes^74^. pCS2-Flag-mTAZ-4SA, pCS2-TEAD-VP16 and pCSP1-Flag-YAP-5SA were digested with a specific restriction enzyme (NotI for Flag-TAZ-4SA and TEAD-VP16, AscI for Flag-YAP-5SA) and transcribed using the SP6 polymerase (AM1340, Lifetechnology). In the overexpression experiments, one cell-stage *Tg(Hsa*.*CTGF:nlsmCherry)*^*ia49*^ embryos, *Tg(kdrl:GFP)* embryos or *Tg(Hsa*.*CTGF:nlsmCherry)*^*ia49*^*/Tg(kdrl:GFP)* double transgenic embryos were injected with 0,2/0,4 pg of TAZ-4SA and TEAD-VP16 mRNAs and 5/10 pg of YAP-5SA mRNA per embryo. To avoid phenotypic alterations associated with RNA toxicity we injected the lowest concentration of constitutively active forms of YAP and TAZ showing biological activity. The injections of the same amount of wild type YAP/TAZ RNA (used as injection control) did not induce induced overexpression effects in zebrafish embryos. Flag-mTAZ-4SA^75^ was subcloned in pME-MCS (Invitrogen) from a pCS2 vector.MultiSite Gateway LR recombination reaction was performed to recombine the obtained pME-TAZ-4SA, the p5E-fli1a and the p3E-polyA (Invitrogen) into the Tol2 destination vector pDestTol2CG2 (Invitrogen). 20–40 pg of the recombined Tol2 destination vectors were co-injected into one cell-stage *Tg(kdrl:GFP)* zebrafish embryos. The effect of Yap1/Taz transient overactivation in the endothelium by TAZ-4SA expression was analysed at 32 hpf by confocal microscopy. Mkate-CAYAP1 plasmid^53^ was injected into one cell-stage *Tg(kdrl:GFP)* embryos that were analysed by confocal microscopy at 32 hpf.

### Image acquisition and analysis

The fluorescence was visualized using 488 nm (for GFP) and 561 nm (for mCherry) lasers and 20x or 40x immersion objectives (Nikon).

Fluorescence quantification of the images acquired either with the conventional fluorescence or the confocal microscope was carried out using Fiji software, by quantifying the fluorescent signal as integrated density as described elsewhere^76^. For the quantification of the reporter signal specific of the endothelium, *Hsa*.*CTGF*:nlsmCherry reporter fluorescence was acquired with the confocal microscope together with the *kdrl*:GFP fluorescence in 32 hpf double transgenic embryos. In order to isolate the reporter expression in the endothelium, Fiji software was used to filter the mCherry signal using the kdrl:GFP as a mask. For each embryo, a confocal Z-stack projection was realized with the obtained filtered images, and then the fluorescent signal was quantified.

### Generation of *yap1* and *taz* mutants

The generation of the *taz*^*bns35*^ mutant line (a.k.a *wwtr1*^*bns35*^) was described elsewhere^48,49^. To generate the *yap1*^*bns19*^ mutant line, a specific single guide RNA (sgRNA) 5′-ACC TCA TCG GCA CGG AAG GG; was designed (crispr.mit.edu) and cloned into the pT7-gRNA vector (Addgene plasmid #46759). yap1-sgRNA *in vitro* transcription was performed with the MEGAshortscript T7 transcription kit (Ambion) using as template the pT7-gRNA vector linearized with BsmBI. 100 pg of sgRNA and 150 pg of *CAS9* mRNA (obtained from the Addgene plsmid #46757) were co-injected into one-cell stage AB embryos. The *yap1*^*bns19*^ allele is a 41 bp deletion. Genotyping primer pair for *yap1*^*bns19*^ is 5′- CTG TTT GTG GTT TCT GAG GGG-3′ and 5′-TGA GAA AGC TGC CAG ACT CA-3′. Genotyping primer pair for *taz*^*bns35*^ is 5′-TTT GTT GTG CAG TCA CAT TGA G-3′ and 5′-GAG GGC GTC ATG CTC TTC-3′. These alleles can be genotyped by standard PCR and resolved with gel electrophoresis (Fig. S7). The heterozygotes were crossed to *Tg(Hsa*.*CTGF:nlsmCherry)*^*ia49*^, *TgBAC(etv2:EGFP)*^*ci1*^ and *Tg(−5*.*2lyve1b:DsRed)*^*nz101*^ for analyses.

### Quantifying *Hsa*.*CTGF* reporter signal in the endothelium of *yap1/taz* mutants

An incross of *yap1*^+*/*−^*;taz*^+*/*−^ fish were performed to obtain embryos for this experiment. Double homozygous mutants die by 30 hpf and are excluded from analysis. Remaining siblings that are *TgBAC(etv2:EGFP)* and *Tg(Hsa*.*CTGF:nlsmCherry)* positive were embedded in 1% low melting agarose and images were acquired with spinning disk confocal microscope (25x objective) at 48 hpf. Image analyses were done with Imaris software. Firstly, EGFP positive cells were selected to delineate endothelial cell nuclei expressing mCherry. Only endothelium on the side of the embryo closest to the objective lens was analyzed. For each nucleus, average signal intensity of mCherry channel was normalized to average signal intensity of EGFP. The normalized values for all selected nuclei were averaged per animal. The calculation can be summarized by the following equation:

$${X}_{j}=\frac{{\sum }_{i=1}^{n}\frac{mcherr{y}_{ij}}{EGF{P}_{ij}}}{n}$$

where X_j_ is the readout for fish j = 1…N; mCherry and EGFP are average intensities of respective channels for nuclei i = 1…n.

### Characterization of the PCV phenotype

*yap1*^−/−^*;taz*^+*/*−^ embryos and randomly sampled siblings were embedded in 1% low melting agarose for image acquisition with an LSM800 confocal microscope (25x objective) at 30 and 48 hpf. The orthogonal projection function on the Zen software was utilized to obtain transverse sections of the trunk. For cryosection of 72 hpf, *yap1*^−/−^*;taz*^+*/*−^ larvae and randomly sampled siblings animals, were fixed with 4% PFA and genotyped using a small piece of the tail. Larvae positive for the *TgBAC(etv2:EGFP)* transgene were selected. Only *yap1*^−/−^*;taz*^+*/*−^ and WT sibling larvae were embedded in OCT using standard procedures. Each section is 12 μm thick. Sections were permeabilized with 0.1% Triton X-100 followed by blocking with 5% sheep serum. Standard immunohistochemistry was performed with anti-EGFP antibody (GFP-1020, Aves Labs, Portland, OR) and Alexa-568 conjugated phalloidin (A12380, Thermo Fisher). Sections were counterstained with DAPI and imaged with LSM800.

### Characterizing cranial and hyaloid vasculature phenotype in yap1 mutants

Scoring of the cranial vasculature phenotypes in yap1 mutants (obtained from an incross of *yap1* heterozygous adults) was performed blind under a Nikon SMZ25 stereomicroscope. Images of the cranial vasculature in 72 hpf *yap1* mutants (Figure S8A) were obtained with an LSM700 confocal microscope (20x objective). Images of hyaloid vessels of both eyes from 5 dpf animals were taken with spinning disk confocal microscope (40x objective) from the dorsal side of the eye (Fig. S8B).

### Luciferase assays

Luciferase assays were performed in MDA-MB-231 cells with the novel YAP1/TAZ-responsive reporter *Hsa*.*CTGF*-Lux. The −200/+27-*CTGF* promoter fragment was cloned in the pGL3-basic Luciferase Reporter Vector (Promega). Luciferase reporters (50 ng/cm^2^) were transfected together with CMV-β-gal (75 ng/cm^2^) to normalize for transfection efficiency. Each sample was transfected in duplicate and each experiment was repeated at least three times independently. NF2 is Addgene #19701. Latrunculin A was from Santa Cruz. The sequences of the siRNA used in this study are as follows (sense strand sequences are indicated): YAP1 1: GACAUCUUCUGGUCAGAGA dTdT; YAP1 2: CUGGUCAGAGAUACUUCUU dTdT; TAZ 1: ACGUUGACUUAGGAACUUU dTdT; TAZ 2: AGGUACUUCCUCAAUCACA dTdT; control: UUCUCCGAACGUGUCACGU dTdT. YAP1/TAZ siRNA 1 refers to the mix composed of oligos YAP1 1 and TAZ 1; YAP1/TAZ siRNA 2 refers to the mix composed of oligos YAP1 2 and TAZ 2.

### Protein extraction and western blot analysis

For protein extraction 50 embryos per group at 72 hpf were deyolked (deyolking buffer: 55 mM NaCl, 1,8 mM KCl, 1,15 mM NaHCO_3_), washed (washing buffer: 110 mM NaCl, 3,5 mM KCl, 2,7 mM CaCl_2_, 10 mM Tris HCl pH 8,5) and lysed in RIPA buffer (Sigma) supplemented with protease and phosphatase inhibitor cocktail (Sigma). Protein concentrations were determined using the Bradford assay (BioRad) and western blot was performed as previously described^77^.

Anti-YAP1 (63.7) monoclonal antibody (sc-101199) was from Santa Cruz, anti-TAZ (560235) monoclonal antibody was from BD Bioscience.

### Whole-mount *in situ* hybridization (WISH)

Standard WISH procedure was performed as described previously^78^. *yap1*^−/−^*;taz*^+*/*−^ embryos from a cross between *yap1*^+*/*−^*;taz*^+*/*−^ and *yap1*^+*/*−^ fish were identified by their notochord or tail phenotype (see above) and fixed with 4% PFA at 24 and 32 hpf. In parallel, WT embryos from WT fish crosses were fixed with 4% PFA at 24 and 32 hpf. Both *yap1*^−/−^*;taz*^+*/*−^ and WT embryos at each developmental stage were mixed into the same reaction tube after Proteinase K permeabilization. Images were acquired with Nikon SMZ25 stereomicroscope followed by genotyping. The primers used to synthesize probes for *efnb2a* and *mrc1a* are found in Table S1.

### Statistical analyses

In Figs 2, 6, S4 and S7 data are presented as mean ± SEM and statistical comparison between groups were performed using a two-tailed Student’s t-test. Statistical analyses were carried out with Prism software (GraphPad). Statistical tests for Fig. 5C,D were performed using Poisson regression with glm function in R. The cranial vasculature phenotype (Fig. S8A’) has been evaluated using a chi-square test. The number of hyaloid vessel (Fig. S8B’) was tested using standard Student T Test. Boxplots were generated with *ggplot2* package in R.

## Electronic supplementary material

Supplementary figures
