# Supplementary material for: Zebrafish mutants and TEAD reporters reveal essential functions for Yap and Taz in posterior cardinal vein development
Source: Sci Rep. 2018 Jul 5;8:10189. doi: 10.1038/s41598-018-27657-x (PMC6033906; doi:10.1038/s41598-018-27657-x)
Supplement: Supplementary file 1 — Supplementary figures [file 41598_2018_27657_MOESM1_ESM.pdf]

Zebrafish mutants and TEAD reporters reveal essential functions for Yap and Taz in posterior cardinal vein development

Matteo Astone[1], Jason Kuan Han Lai[2], Sirio Dupont[3], Didier Y. R. Stainier[2], Francesco Argenton*[1], Andrea Vettori*[1].

[1]University of Padova, Department of Biology. Padova Italy.

[2]Max Planck Institute for Heart and Lung Research, Bad Nauheim, Germany.

[3]University of Padova, Department of Molecular Medicine. Padova Italy.

*Corresponding authors Francesco.argenton@unipd.it, andrea.vettori@unipd.it

Figure S1

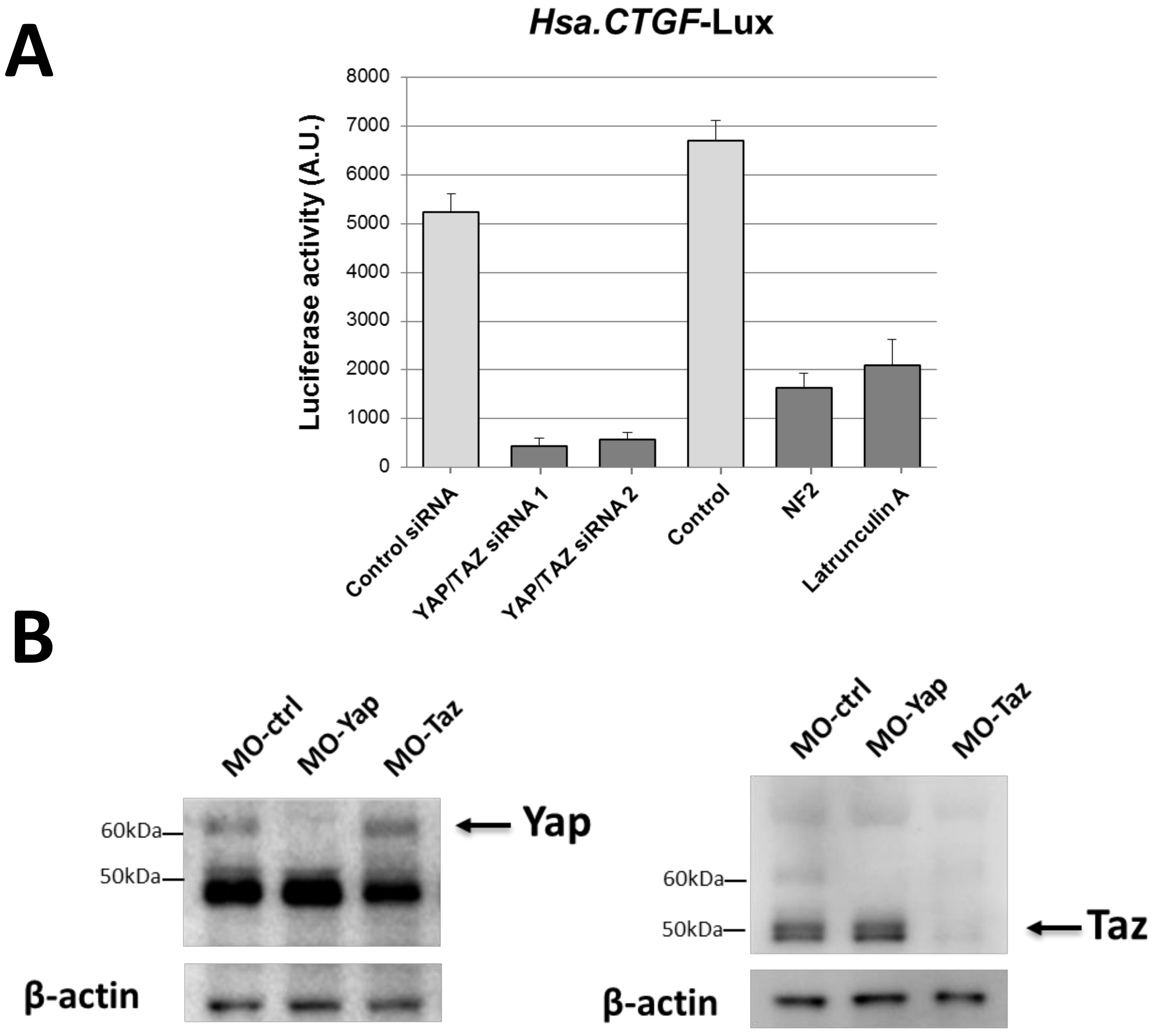

Figure S1. Validation of the *Hsa.CTGF*-based YAP1/TAZ reporter construct and the Yap1/Taz morpholinos. (A) The *Hsa.CTGF*-Lux reporter is specific for YAP1/TAZ activity. MDA-MB-231 cells, expressing high levels of TAZ, were transfected with the indicated siRNAs or plasmids (control is empty vector, NF2 is NF2 expression plasmid), or treated with latrunculin A. The panels display the results of luciferase tests with the *Hsa.CTGF*-Lux. YAP1/TAZ siRNAs dramatically downregulates the reporter with respect to control siRNA. Luciferase expression was also significantly reduced after either NF2-mediated Hippo pathway activation or latrunculin-mediated mechanical pathway inhibition. (B) Western blot analysis of morpholino-mediated Yap1 and Taz knockdown.

Figure S2

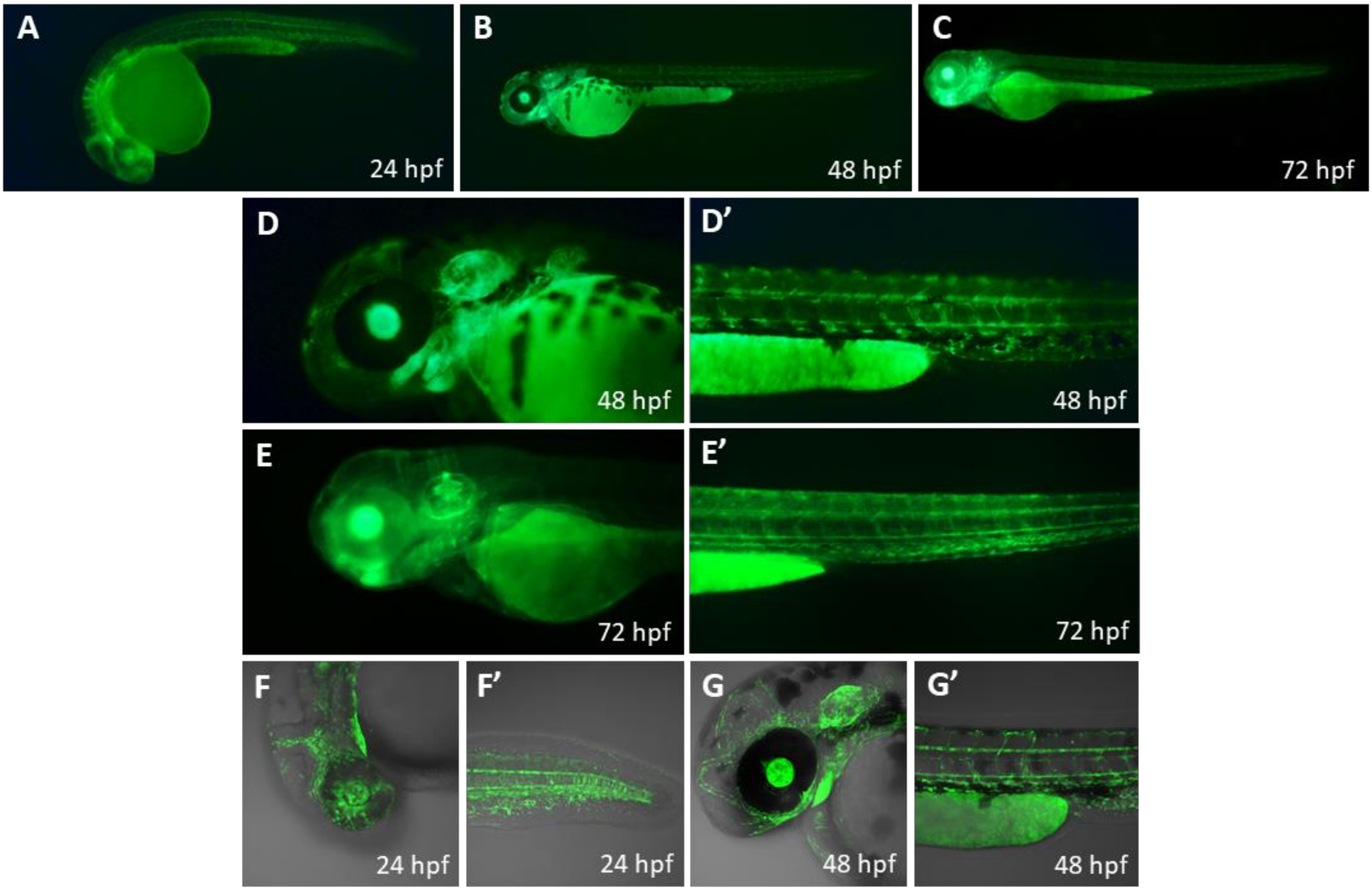

Figure S2. Overview of the *Tg(Hsa.CTGF:eGFP)* reporter expression. *In vivo* fluorescent microscope **(**A-E') and confocal images (F-G') of *Tg(Hsa.CTGF:eGFP)* larvae at 24, 48 and 72 hpf.

Figure S3

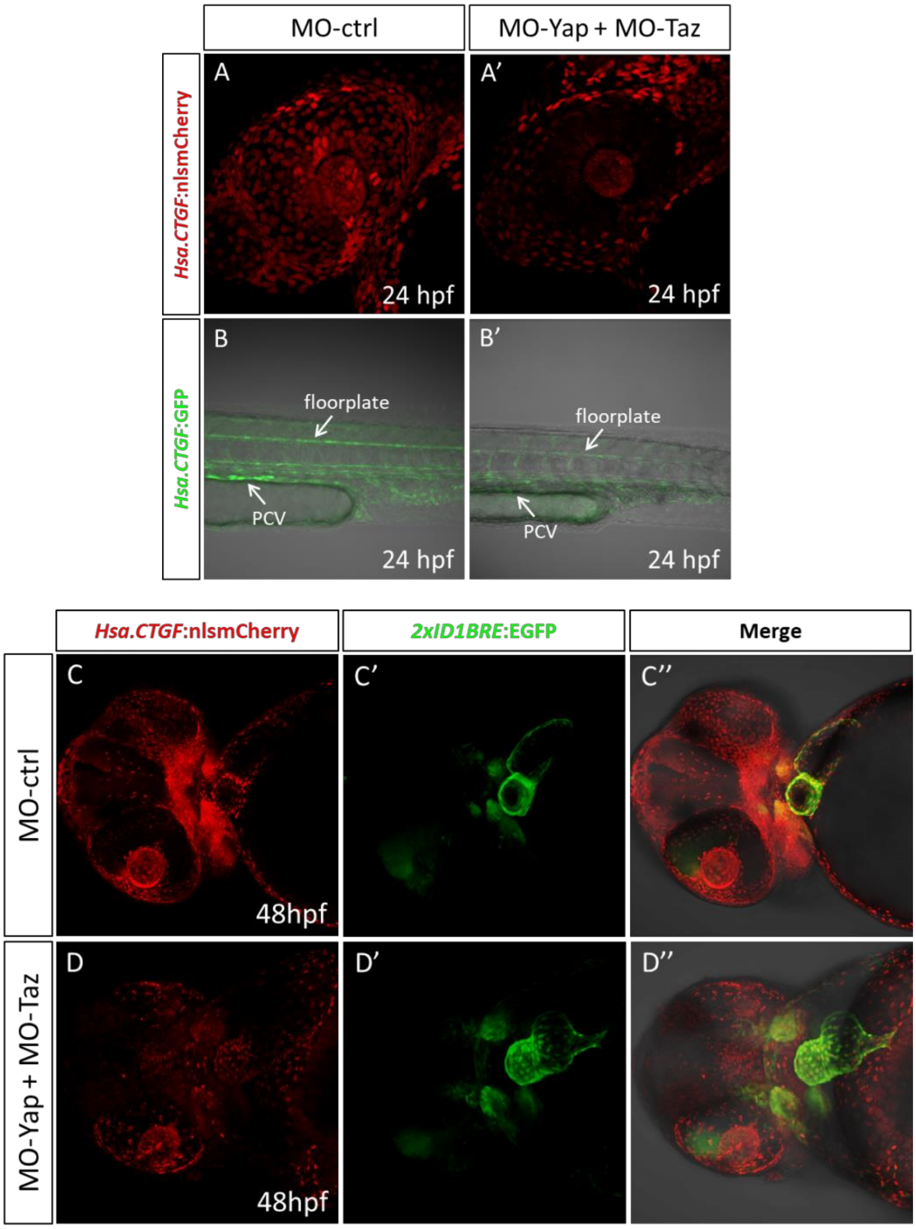

Figure S3. ***Hsa.CTGF*****-based reporter signal is dependent on Yap1/Taz in specific tissues.** (A,A') Confocal Z-stack projections of the eye of *Tg(Hsa.CTGF:nlsmCherry)*^{ia49} embryos injected with Yap1/Taz or control morpholinos. Yap1/Taz knockdown considerably reduces the reporter signal in the eye. (B,B') Confocal Z-stack projections of the trunk of *Tg(Hsa.CTGF:eGFP)*^{ia48} embryos injected with Yap1/Taz or control morpholinos. The decrease of the reporter signal upon Yap1/Taz knockdown is clearly visible in the floorplate and the PCV. (C-D'') Confocal Z-stack projections of the heart region of *Tg(Hsa.CTGF:nlsmCherry)*^{ia49}/*Tg(2xID1BRE:GFP)*^{ia17} embryos injected with Yap1/Taz or control morpholinos. *Tg(2xID1BRE:GFP)*^{ia17} is used here to highlight the heart structure. Yap1/Taz knockdown reduces the Yap1/Taz reporter signal in the heart. PCV: posterior cardinal vein.

Figure S4

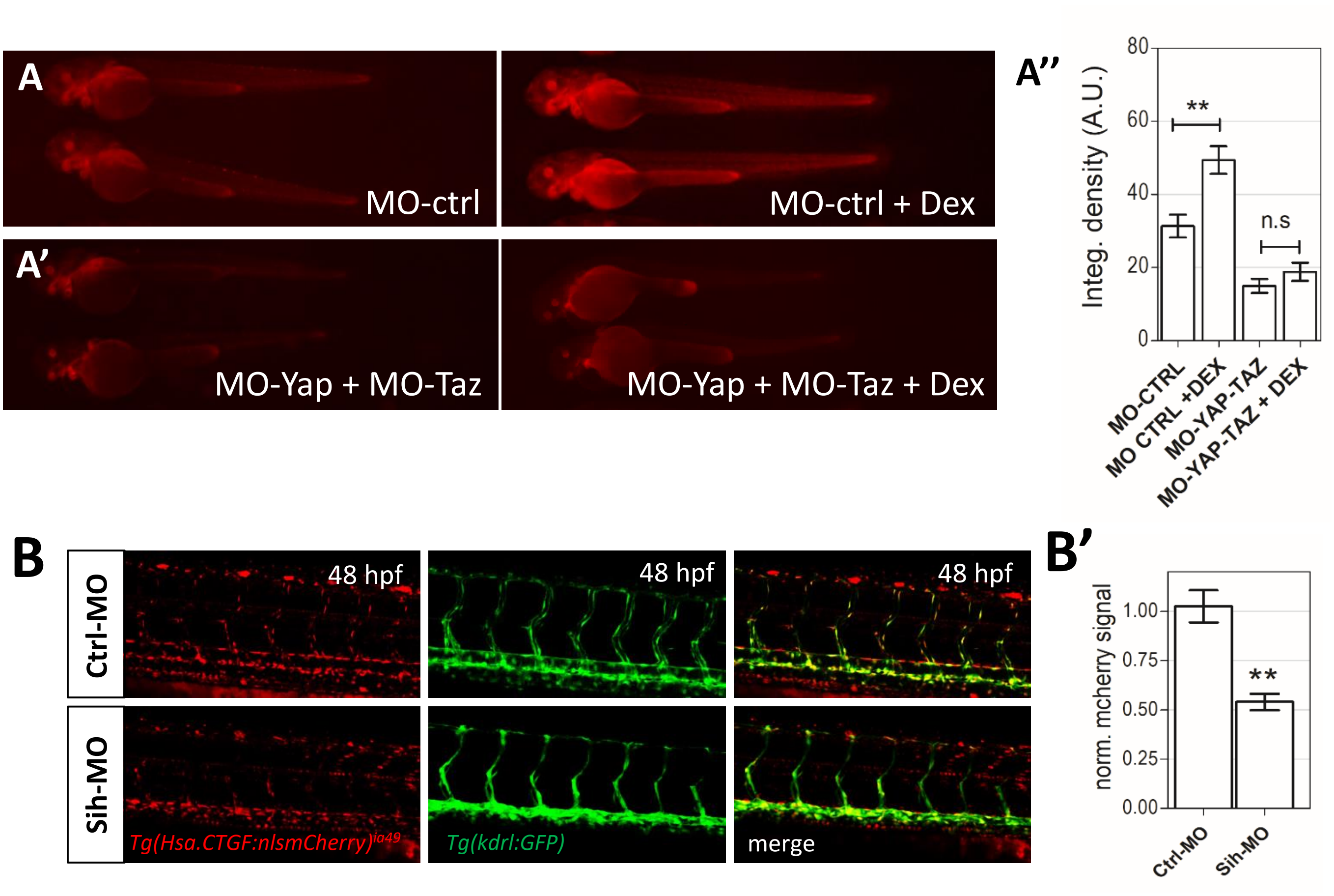

Figure S4. *Hsa.CTGF*-based Yap1/Taz zebrafish reporter is modulated by glucocorticoids and blood flow. (A,A') Representative image of *Tg(Hsa.CTGF:nlsmCherry)*^{ia49} transgenic embryos injected with Yap1/Taz or control morpholinos alone or combined with 25 µM Dexamethasone (DEX) from 24 hpf to 48 hpf. Dex-activation of *Hsa.CTGF* transgene is maintained in control morpholino-injected embryos and ablated in Yap/Taz morphants. (A'') Average values of fluorescence integrated density calculated for treated embryos and controls. For each group a minimum of 10 embryos were analyzed.

(B) Representative confocal images of *Tg(Hsa.CTGF:nlsmCherry)*^{ia49}*/tg(kdrl:GFP)* double transgenic embryos injected with control or *sih* morpholinos at 48 hpf. In *sih* morphants, the activity of the hCTGF reporter in the endothelium is reduced. (B') Average values of fluorescence integrated density calculated for treated embryos (n=5) and controls (n=6). The mCherry fluorescence was measured in the endothelial cells and normalized for the GFP fluorescence used as internal standard. A.U., arbitrary units. ** = p<0.01.

Figure S5

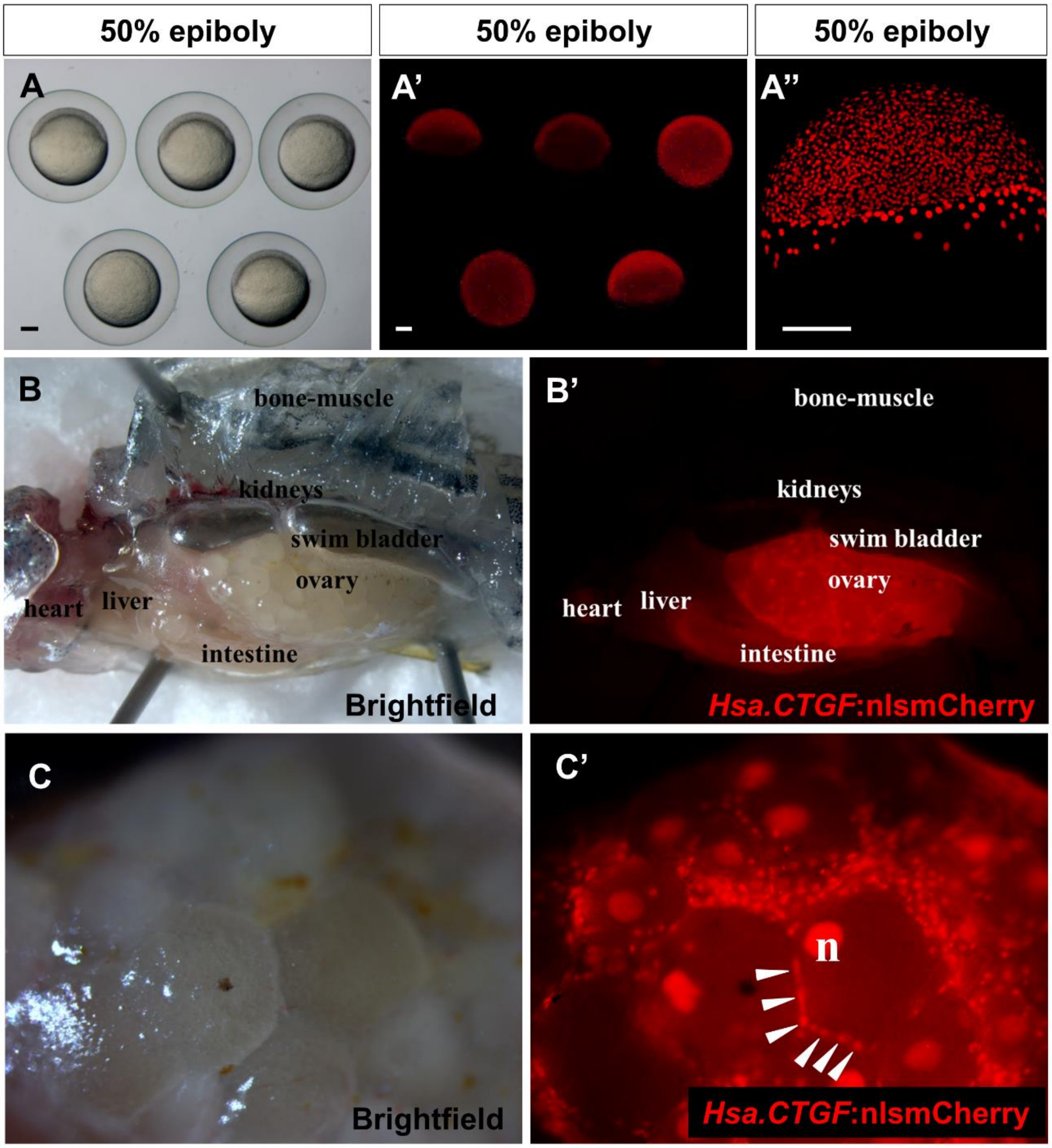

Figure S5. Yap1/Taz reporter expression is activated maternally. (A-A'') 50%-epiboly stage embryos from *Tg(Hsa.CTGF:nlsmCherry)^{ia49}* female showing the ubiquitous maternally activated Yap1/Taz reporter fluorescence. Brightfield (A), fluorescence (A') and confocal (lateral view) (A'') images are shown. (B-B') Internal organs of a *Tg(Hsa.CTGF:nlsmCherry)^{ia49}* adult female. A strong reporter fluorescence is detected especially in the ovary. (C-C') Zoomed view of the ovary. Transgene signal is visible in the nuclei (n) of the oocytes at advanced maturation stages and in the accompanying follicle cells, arranged in a single layer adjacent to the oocytes (arrowheads). Scale bar: 100 µm.

Figure S6

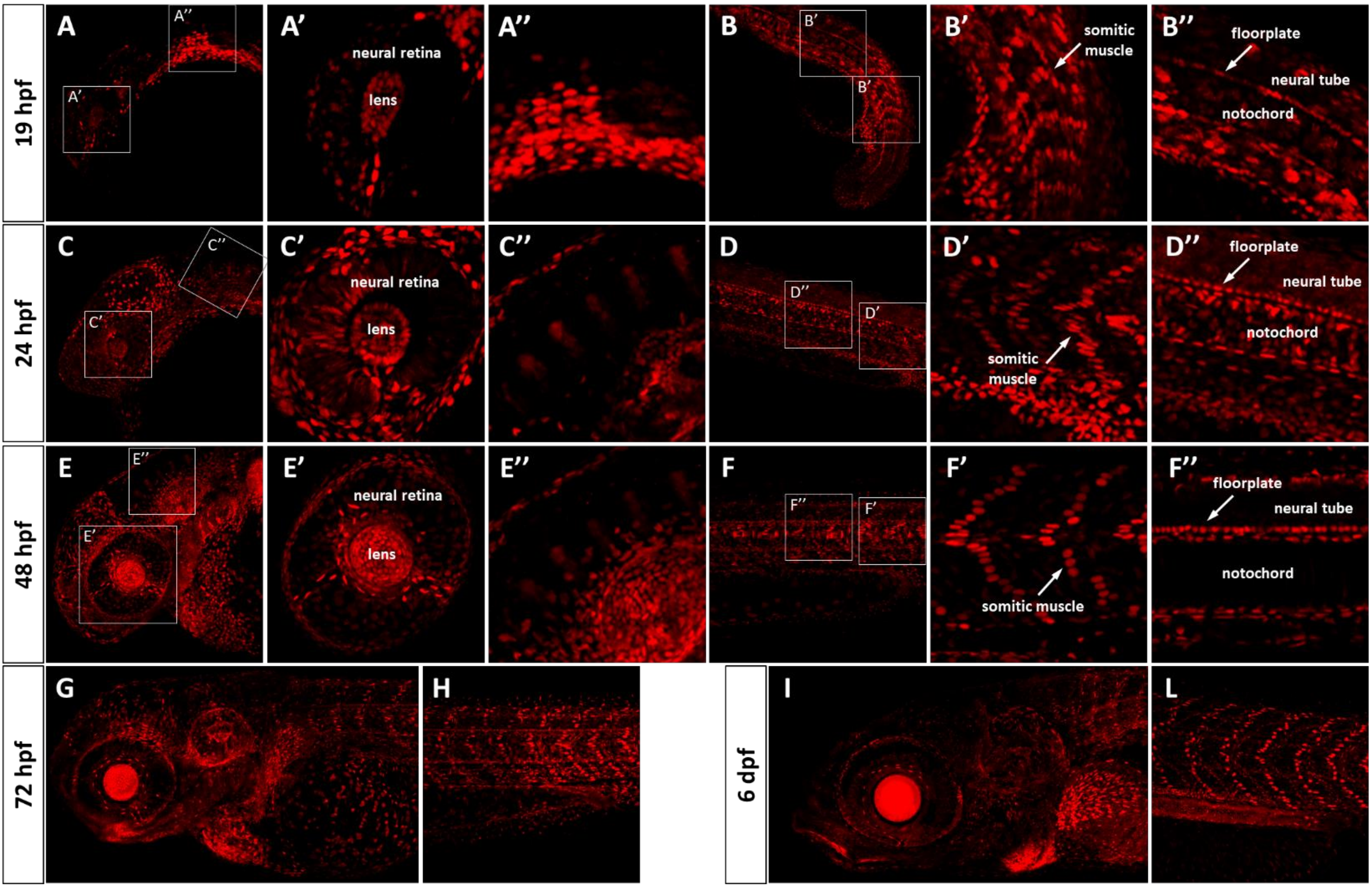

Figure S6. Time series of Yap1/Taz reporter expression during embryonic and early larval stage. Confocal Z-stack projections of a *Tg(Hsa.CTGF:nlsmCherry)*^{ia49} fish at 19 hpf (A-B''), 24 hpf (C-D''), 48 hpf (E-F''), 72 hpf (G-H), and 6 dpf (I-L). (A, C, E, G, I) Full z-stack projection of the rostral region of the embryo/larva. (B, D, F, H, L) Full z-stack projection of the trunk of the embryo/larva. Partial z-stack projection limited to the optic cup (A', C', E'), the rhombencephalon (A'', C'', E''), the somitic muscles (B', D', F'), and the notochord region (B'', D'', F'') are reported in the magnified views. Relevant cell types where the reporter is strongly activated are highlighted at each time point. For the details regarding the reporter protein expression in the different tissues refer to the text and to Figures 3 and 4.

Figure S7

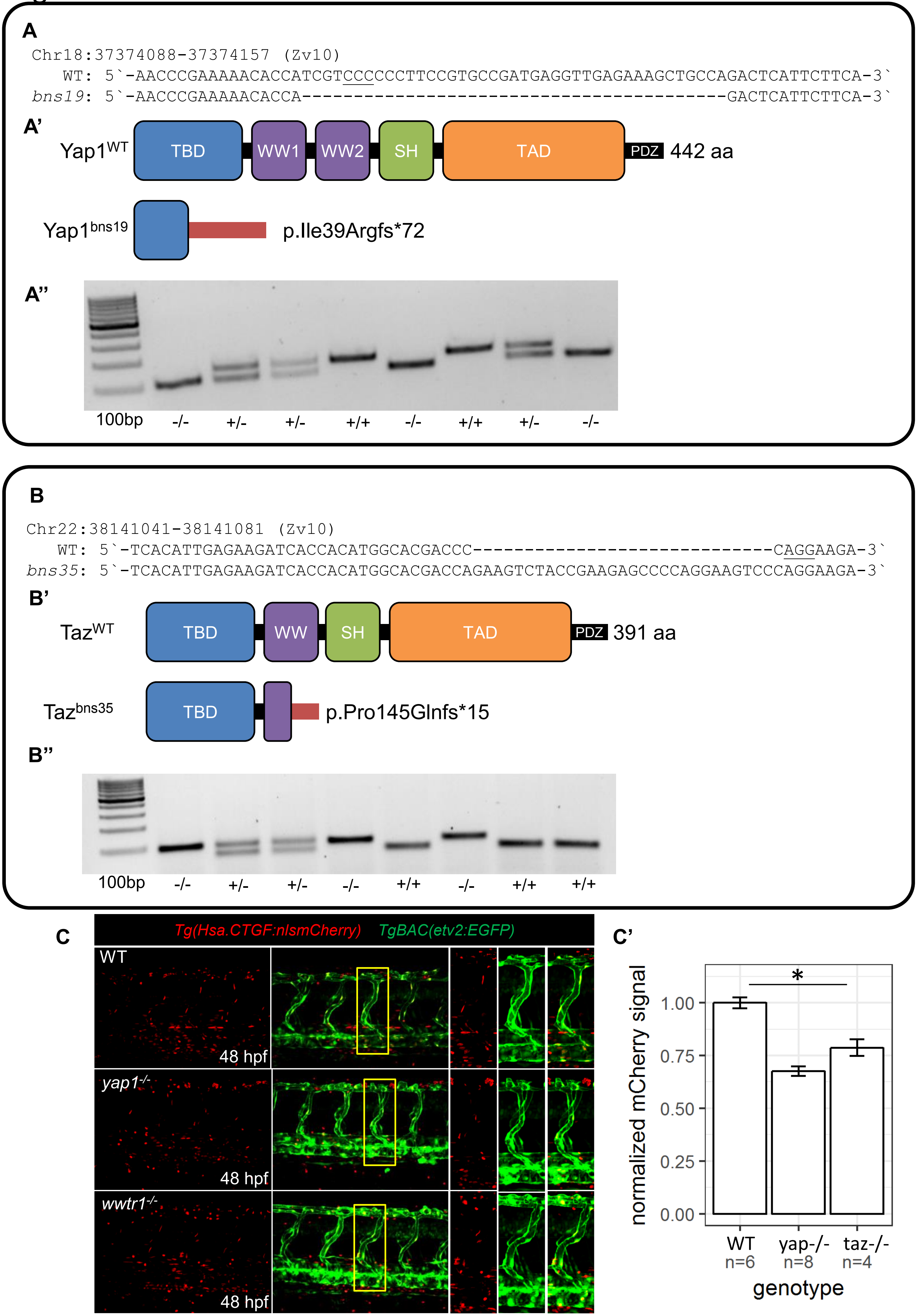
A
Chr18:37374088-37374157 (Zv10)
WT: 5`-AACCCGAAAAACACCATCGTCCCCCCTTCCGTGCCGATGAGGTTGAGAAAGCTGCCAGACTCATTCTTCA-3`
bns19: 5`-AACCCGAAAAACACCA-------------------------------------------GACTCATTCTTCA-3`
A'
Yap1WT
TBD
WW1
WW2
SH
TAD
PDZ
442 aa
Yap1bns19
p.Ile39Argfs*72
A''
100bp
-/-
+/-
+/-
+/+
-/-
+/+
+/-
-/-
B
Chr22:38141041-38141081 (Zv10)
WT: 5`-TCACATTGAGAAGATCACCACATGGCACGACCC------------------------------CAGGAAGA-3`
bns35: 5`-TCACATTGAGAAGATCACCACATGGCACGACCAGAAGTCTACCGAAGAGCCCCAGGAAGTCCCAGGAAGA-3`
B'
TazWT
TBD
WW
SH
TAD
PDZ
391 aa
Tazbns35
TBD
p.Pro145Glnfs*15
B''
100bp
-/-
+/-
+/-
-/-
+/+
-/-
+/+
+/+
C
Tg(Hsa.CTGF:nlsmCherry)
TgBAC(etv2:EGFP)
WT
48 hpf
yap1-/-
48 hpf
wwtr1-/-
48 hpf
C'
normalized mCherry signal
1.00
0.75
0.50
0.25
0.00
*
WT
n=6
yap-/-
n=8
taz-/-
n=4
genotype

Figure S7. CRISPR targeted mutagenesis of the zebrafish *yap1* and *taz* loci. Single guide RNAs (sgRNAs) were designed to target the first exon of *yap1* and the second exon of *taz*. Sequence alignments between WT and mutant sequences for $yap1^{bns19}$ (A) and taz^{bns35} (B). The underlined nucleotides are the PAM sequences. (A' and B') These indel mutations result in a frameshift and are predicted to encode a truncated protein product as represented in the cartoon. (A'' and B'') The genotyping of these alleles can be performed by simple PCR followed by resolution of the WT and mutant amplicons by gel electrophoresis. (C) Representative confocal images of *Tg(Hsa.CTGF:nlsmCherry);TgBAC(etv2:EGFP)* transgenic embryos in *yap* and *taz* mutant background. Higher magnification images correspond to the region demarcated in yellow. (C') The mCherry fluorescence was measured in the endothelial cells and normalized for the GFP fluorescence used as internal standard. n for each group is indicated. * = $p<0.05$.

Figure S8

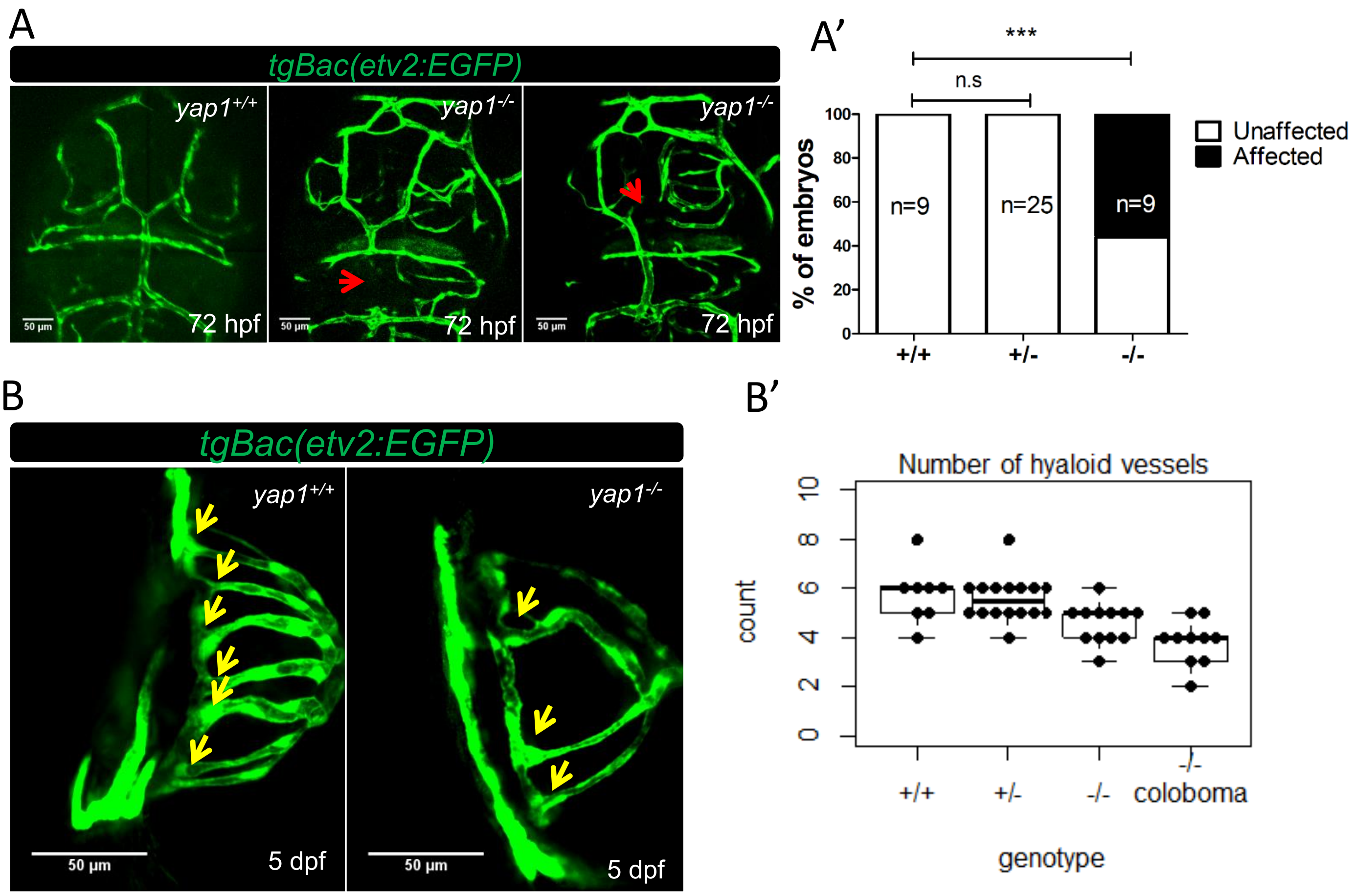

Figure S8. *yap1$^{-/-}$* larvae exhibit cranial and hyaloid vasculature defects. (A) Representative figures of *yap1* mutants showing cranial vasculature defects compared to WT sibling at 72 hpf. The red arrows point to lack of endothelial transgene expression in the mesencephalic vein (MsV) and dorsal longitudinal vein. (A') The cranial vasculature phenotype is partially penetrant. (B) Dorsal view of the eyes of 5 dpf WT and mutant siblings. (B') Number of hyaloid vessels per embryo for each genotype. Each point represents one eye. The number of hyaloid vessels of wild-type embryos is statistically higher in comparison to mutants with ($p<0.01$) or without ($p<0.05$) coloboma by student T test. n for each group is indicated. *** = $p<0.001$.

Figure S9

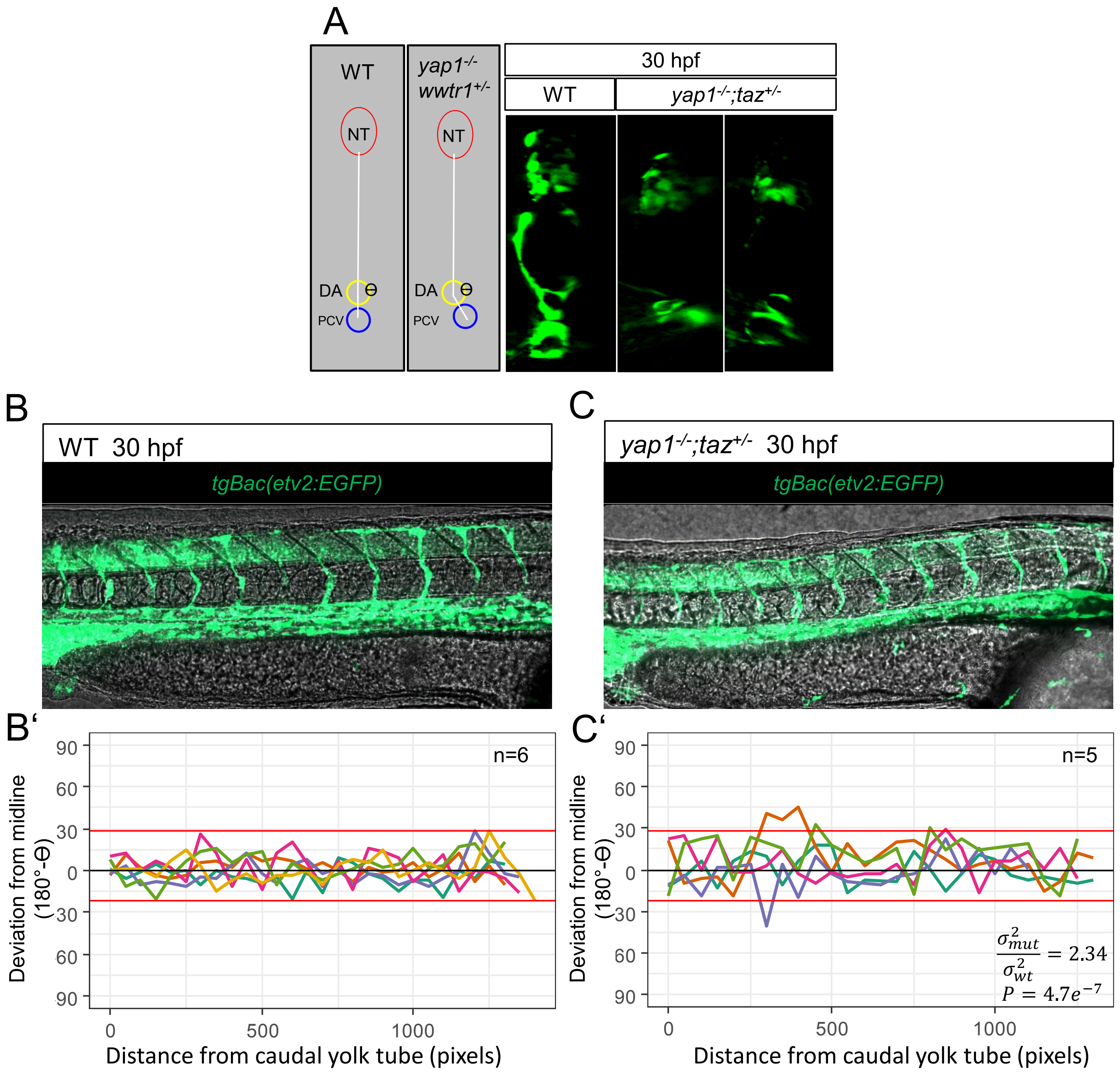

Figure S9. PCV defects in 30 hpf *yap1$^{-/-}$;taz$^{+/-}$* embryos. (A) Transverse sections of trunk to show the relative positions of the neural tube (NT), dorsal aorta (DA) and posterior cardinal vein (PCV) in tgBac(etv2:EGFP). (B, C) Maximum intensity projections from the right side. (B', C') Quantification of PCV deviation was performed as described in Figure 5B-D. σ_{mut}^2: variance of (180° - ϴ) in *yap1$^{-/-}$;taz$^{+/-}$* animals; σ_{WT}^2: variance of (180° - ϴ) in WT animals. n for each group is indicated.

Figure S10

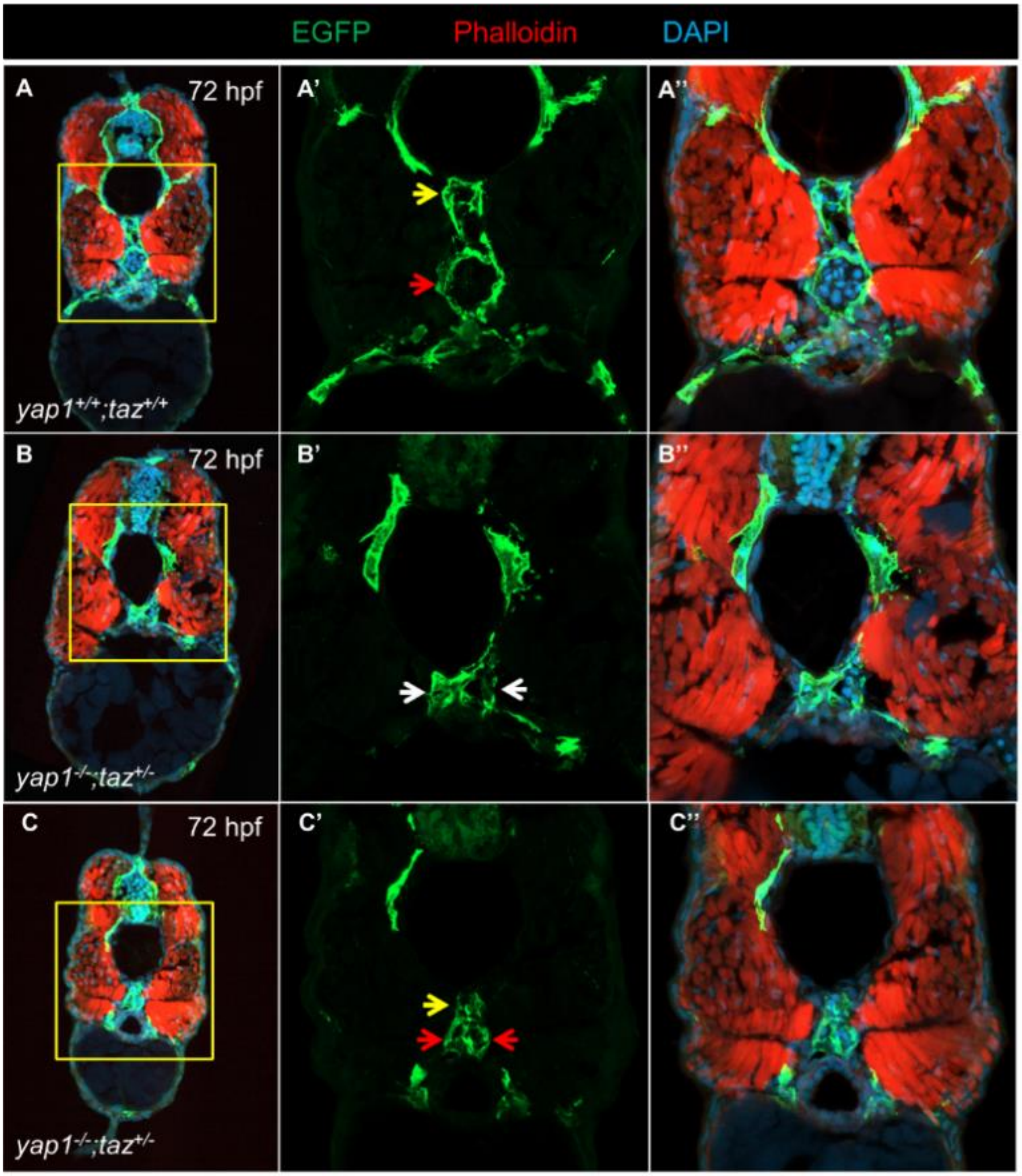

Figure S10. PCV defects in 72 hpf *yap1$^{-/-}$;taz$^{+/-}$* animals. Transverse cryosection of *TgBac(etv2:EGFP)* WT (A) and *yap1$^{-/-}$;taz$^{+/-}$* (B and C) larvae at 72 hpf. (A'-C', A''-C'') High magnification images of regions demarcated in yellow. Yellow arrows: DA; red arrows: PCV; white arrows: DA or PCV.

Supplementary table S1

Table S1. List of primers used for WISH probes		
Gene	Primer Sequences (5' - 3')	Remarks
efnb2a	CATTAACCCTCACTAAAGGGAAGGGGAAGATAAGGGAGATGG	RNA synthesis with T7 enzyme. PCR product as template.
	TAATACGACTCACTATAGGGGCGTGTCCATTTTCACACCT	
mrc1a	CATTAACCCTCACTAAAGGGAAGATATCCAGGGGTTCCGAAA	RNA synthesis with T7 enzyme. PCR product as template.
	TAATACGACTCACTATAGGGGGCATGTTCATTCTGTTCGA	
